# Wound-Healing Potential of Rhoifolin-Rich Fraction Isolated from *Sanguisorba officinalis* Roots Supported by Enhancing Re-Epithelization, Angiogenesis, Anti-Inflammatory, and Antimicrobial Effects

**DOI:** 10.3390/ph15020178

**Published:** 2022-01-31

**Authors:** Walaa A. Negm, Aya H. El-Kadem, Engy Elekhnawy, Nashwah G. M. Attallah, Gadah Abdulaziz Al-Hamoud, Thanaa A. El-Masry, Ahmed Zayed

**Affiliations:** 1 Department of Pharmacognosy, Faculty of Pharmacy, Tanta University, Tanta 31527, Egypt; walaa.negm@pharm.tanta.edu.eg; 2 Department of Pharmacology and Toxicology, Faculty of Pharmacy, Tanta University, Tanta 31527, Egypt; aya.elkadeem@pharm.tanta.edu.eg (A.H.E.-K.); thanaa.elmasri@pharm.tanta.edu.eg (T.A.E.-M.); 3 Pharmaceutical Microbiology Department, Faculty of Pharmacy, Tanta University, Tanta 31527, Egypt; engy.ali@pharm.tanta.edu.eg; 4 Department of Pharmaceutical Science College of Pharmacy, Princess Nourah bint Abdulrahman University, P.O. Box 84428, Riyadh 11671, Saudi Arabia; 5 Department of Pharmacognosy, College of Pharmacy, King Saud University, Riyadh 11495, Saudi Arabia; galhamoud@ksu.edu.sa; 6 Institute of Bioprocess Engineering, Technical University of Kaiserslautern, Gottlieb-Daimler-Straße 49, 67663 Kaiserslautern, Germany

**Keywords:** antibiofilm, anti-inflammatory, immunomodulatory, LC-MS/MS, MMP-1, TGF-*β*

## Abstract

A wound is a complicated bioprocess resulting in significant tissue damage, which is worsened by a secondary bacterial infection, commonly *Pseudomonas aeruginosa* and *Staphylococcus aureus*. The goal of our study was to investigate the metabolic profile and possible wound-healing effect of *Sanguisorba officinalis* roots rhoifolin rich fraction (RRF). The LC-ESI-MS/MS analysis of *S. officinalis* roots crude ethanol extract resulted in a tentative identification of 56 bioactive metabolites, while a major flavonoid fraction was isolated by column chromatography and identified by thin-layer chromatography coupled with electrospray ionization/mass spectrometry (TLC-ESI/MS), where rhoifolin was the major component representing 94.5% of its content. The antibiofilm activity of RRF on the mono-species and dual-species biofilm of *P. aeruginosa* and *S. aureus* was investigated. RRF exhibited inhibitory activity on *P. aeruginosa* and *S. aureus* mono-species biofilm at 2× minimum inhibitory concentration (MIC) and 4× MIC values. It also significantly inhibited the dual-species biofilm at 4× MIC values. Moreover, the wound-healing characteristics of RRF gel formulation were investigated. Rats were randomly allocated into four groups (eight rats in each): Untreated control; Blank gel; Betadine cream, and RRF gel groups. Animals were anesthetized, and full-thickness excisional skin wounds were created on the shaved area in the dorsal skin. The gels were topically applied to the wound’s surface daily for 10 days. The results demonstrated that RRF had a promising wound-healing effect by up-regulating the platelet-derived growth factor (PDGF), vascular endothelial growth factor (VEGF), keratinocyte growth factor (KGF), and fibronectin, while metalloproteinase-1 (MMP-1), interleukin-6 (IL-6), IL-1*β*, and nitric oxide (NO) levels were suppressed. It also enhanced the immune staining of transforming growth factor (TGF-*β*) and improved histopathological findings. Furthermore, it displayed an immunomodulatory action on lipopolysaccharide-induced peripheral blood mononuclear cells. Hence, the wound-healing effect of rhoifolin was confirmed by supporting re-epithelization, angiogenesis, antibacterial, immunomodulatory, and anti-inflammatory activities.

## 1. Introduction

Wounds represent a major health problem in which the skin is damaged by numerous overlapping processes such as hemostasis, inflammation, proliferation, and remodeling [1,2]. Wound healing is a multi-step process that requires the cooperation of various cell types such as keratinocytes, fibroblasts, endothelial cells, macrophages, and platelets [3]. Bacteria and fungus can easily contaminate wounds, slowing the healing process. As a result, topical antimicrobials are prescribed in an attempt to limit the possible infection of deeper body tissues and blood circulation, which could lead to sepsis [4]. However, some topical antibiotic preparations may have cytotoxic effects in addition to antibiotic resistance, hindering and complicating wound healing. The use of medicinal plant preparations can exert beneficial effects due to their biocompatibility, wound healing, and antimicrobial properties.

*Pseudomonas aeruginosa* (Gram-negative bacterium) and *Staphylococcus aureus* (Gram-positive bacterium) usually cause biofilm-related infections. These mixed infections are commonly isolated from infected wounds, suppurative otitis media, in addition to indwelling medical devices. The eradication of bacteria that are present in biofilms is very difficult, as the polymer matrix reduces their sensitivity to the antimicrobials as well as the host immune defense [5].

*Sanguisorba officinalis* L. (family Rosaceae) radix has been used in Chinese traditional medicine for thousands of years to cure ailments such as burns, inflammation, scalds, ulcers, eczema, acne, diarrhea, and bleeding [6,7,8,9,10,11]. The main compounds that are partially responsible for the medicinal actions of *S. officinalis* have been identified as triterpenoids, triterpenoid glycosides, flavonoids, lignans, lignosides, polysaccharides, hydrolyzable tannins, and monoterpene glycosides [8,12,13,14,15,16,17]. Most previous studies have focused on the pharmacological effects of *S. officinalis* associated with its terpenoid contents [7,18,19,20].

Here, we investigated the phytochemical profiling of *S. officinalis* including phenolic acids, alkaloids, flavonoids, triterpenoids, fatty acids, and other compounds in addition to focusing on the major flavonoids fraction, including rhoifolin-rich fraction (RRF). The potential beneficial activities of RRF as a topical treatment in supporting wound healing have not been investigated yet. So, the major aim of our study is to investigate the wound-healing potential of RRF in vivo. In addition, another aim is to investigate the impact of RRF on *P. aeruginosa* and *S. aureus* mono-species and dual-species biofilms in vitro *and* the immunomodulatory effect of RRF in vitro on lipopolysaccharides-induced peripheral blood mononuclear cells (PBMCs).

## 2. Results

### 2.1. LC-ESI-MS/MS Analysis of S. officinalis Extract

The positive and negative ionization mode of LC-ESI-MS/MS analysis of *S. officinalis* roots ethanol extract resulted in the detection of a total of 63 bioactive metabolites, including 56 tentatively identified belonging mainly to flavonoids and triterpenoids, in addition to seven unknown metabolites, as shown in Table 1. The TIC showed a high intensity for compounds between 11–13 and 11–16 min for negative and positive mode, respectively. Such a pattern resulted in hindering the intensity in the mid-polarity area (Figures S1 and S2).

Additionally, Table 1 showed the metabolic annotation demonstrating the corresponding molecular formula, RT (min), which was the used method for annotation either from LC-ESI-MS/MS libraries or previous literature and MS^2^ main fragments.

The results of metabolic profiling revealed that luteolin-6-*C*-glucoside (P17), isookanin-7-glucoside (P20), and rhoifolin (P26) were among the major peaks detected in negative mode, while 1-*O*-*β*-_D_-glucopyranosyl sinapate (P5), catechin (P15), and 3,3′,4′,5-tetrahydroxy-7-methoxyflavone (P32) were among those in the positive counterpart (Table 1).

#### 2.1.1. Flavonoids

Despite flavonols being the only class of flavonoids detected in previous research [9], the current research showed numerous classes of flavonoids, flavonoid glycosides, and chalcones, as shown in Table 1. Examples included flavones represented by rhoifolin, apigenin, luteolin, and its 6-*C*-glucoside, isorhamnetin-3-*O*-glucoside, and 3,3′,4′,5-tetrahydroxy-7-methoxyflavone, in addition to flavanone glycoside, i.e., isookanin-7-glucoside, eriodictyol-7-*O*-glucoside, and naringenin-7-*O*-glucoside. Moreover, flavonol glycosides were detected, including quercetin-3-_D_-xyloside, quercetin-3-arabinoside, kaempferol-3-glucuronide, and kaempferol-3-*O*-*α*-_L_-arabinoside. Other flavonoid-related metabolites were identified, i.e., flavan-3-ol, as catechin and epicatechin. They are recognized as the building blocks of proanthocyanidin (e.g., procyanidin B2 and procyanidin C1).

#### 2.1.2. Triterpenoids

Similarly, several triterpenoids were annotated in the current research (Table 1) based on their fragmentation pattern in agreement with previous literature [17]. Among them were ziyuglycoside I, lup-12-en-15*α*,19*β*-diol-3,11-dioxo-28-oic acid, and euscaphic acid or arjunic acid, which have been isolated previously in *S. officinalis* by Kim et al. [8], Zhang et al. [11], and Kim et al. [16], respectively. Such triterpenoids demonstrated potential bioactivities, including anti-inflammatory, antioxidant, cosmeceutical, and anti-bacterial effects.

As revealed in TIC (Figures S1 and S2), the high metabolite abundances were shown to be corresponding to triterpenoids, i.e., retention times between 11 and 16 min. Thus, they were confirmed to be major constituents in the root extract of *S. officinalis*.

### 2.2. Characterization of the Rhoifolin Rich Fraction RRF

**Figure 1:**
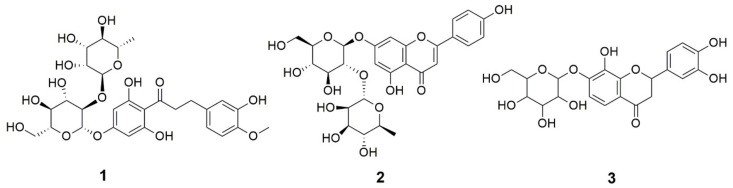
The chemical structures of compounds tentatively identified in rhoifolin rich fraction (RRF) based on TLC-ESI/MS results, i.e., neohesperidin dihydrochalcone (**1**) (% peak area = 5.2), apigenin 7-*O*-neohesperidoside (rhoifolin) (**2**) (% peak area = 94.5), and isookanin-7-glucoside (flavanomarein) (**3**) (% peak area = 0.3).

Three flavonoids were detected in this fraction as shown by the total ion chromatogram (TIC) of thin layer chromatography coupled with electrospray ionization/mass spectrometry (TLC-ESI/MS), as shown in Figure S3. The results showed that compound **2** (RT = 0.49 min) was apigenin 7-*O*-neohesperidoside (rhoifolin), which represented the most abundant flavonoid with a percentage peak area = 94.5, while compound **1** (RT = 0.28 min) and **3** (RT = 1.37 min) were neohesperidin dihydrochalcone and isookanin-7-glucoside (flavanomarein) with the percentage peak area of 5.2 and 0.3, respectively. The mass and other spectroscopic identities of these compounds were consistent with previously published data [22,23,24]. Since rhoifolin was the major flavonoid, the fraction was named rhoifolin rich fraction (RRF). Figure 1 presents the chemical structures of these compounds, while the mass spectra are displayed in Figure S4.

### 2.3. In Vitro Activities

A total of 15 *P. aeruginosa* and 26 *S. aureus* isolates were obtained from wound infections from patients in Tanta University Hospital. The minimum inhibitory concentration (MIC) values of RRF against the tested bacterial isolates were identified using the broth microdilution method to determine the impact of RRF on the planktonic bacteria. The MIC values of RRF varied from 64 to 512 µg/mL. About 26.67% and 15.38% of *P. aeruginosa* and *S. aureus* isolates, respectively, were strong biofilm producers by crystal violet assay. The values of MICs of RRF against the tested isolates and the level of their biofilm-forming ability are shown in Tables S1 and S2.

#### 2.3.1. Antibiofilm Activity

**Figure 2:**
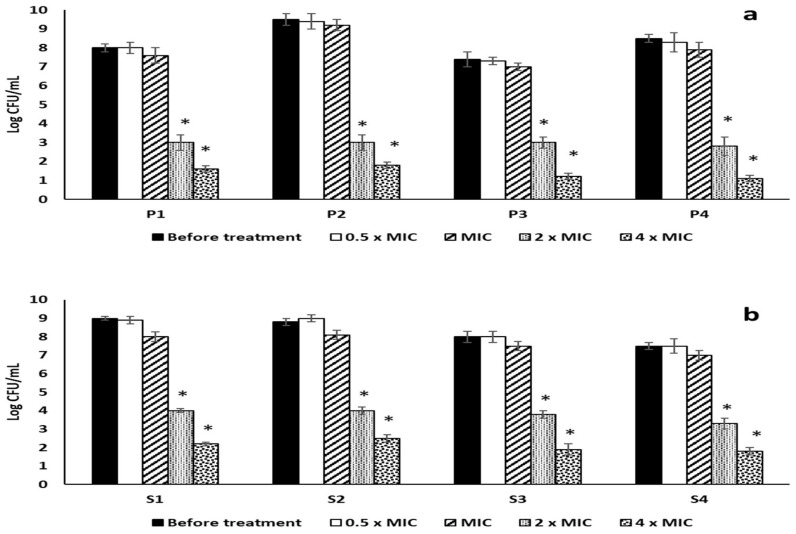
Impact of RRF on mono-species biofilms of (**a**) *P. aeruginosa* and (**b**) *S. aureus* isolates. The artistic symbol (*) represents a significant decrease in biofilm formation (*p* < 0.05).

**Figure 3:**
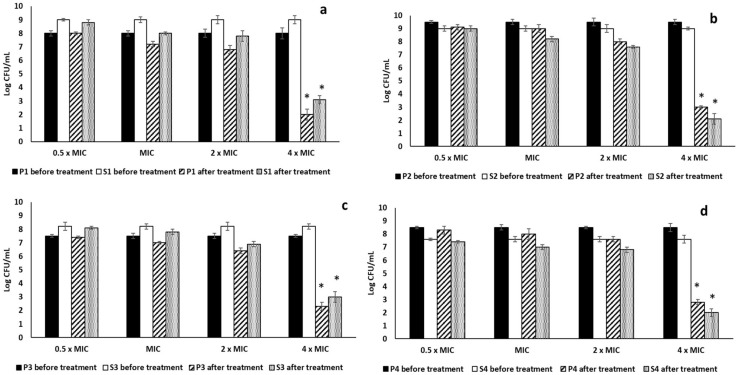
Impact of RRF on dual-species biofilms of (**a**) P1 + S1, (**b**) P2 + S2, (**c**) P3 + S3, and (**d**) P4 + S4. The artistic symbol (*) represents a significant decrease in biofilm formation (*p* < 0.05).

The antibiofilm activity of RRF was evaluated against mono-species biofilms and dual-species biofilms of four *P. aeruginosa* and four *S. aureus* isolates, which showed a strong biofilm-forming ability by crystal violet assay. RRF showed MIC values of 128, 64, 256, and 256 μg/mL against the four selected *P. aeruginosa* isolates P1, P2, P3, and P4, respectively. In addition, RRF showed MIC values of 128, 64, 256, and 256 μg/mL against the four selected *S. aureus* isolates S1, S2, S3, and S4, respectively. The values of 2× MIC and 4× MIC showed a considerable reduction (*p* < 0.05) in the formation of the mono-species biofilms. On the other hand, the 4× MIC value of RRF showed a significant decrease (*p* < 0.05) in the dual-species biofilms formation compared to the non-treated dual-species, as shown in Figure 2 and Figure 3.

#### 2.3.2. Immunomodulatory Activity

##### MTT Assay

**Figure 4:**
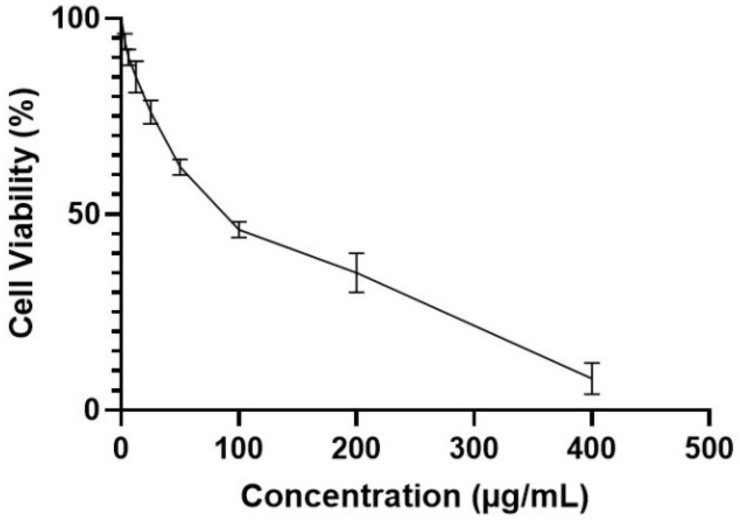
Cytotoxicity of RRF on PBMCs by MTT assay. The IC_50_ was determined by three independent tests.

The effect of RRF on the viability of PBMCs was evaluated at concentrations of 3.125, 6.25, 12.5, 25, 50, 100, 200, and 400 μg/mL. The IC_50_ of RRF against PBMCs was determined at 80.3 ± 0.91, as exhibited in Figure 4.

##### Quantitative Real-Time Polymerase Chain Reaction (qRT-PCR)

**Figure 5:**
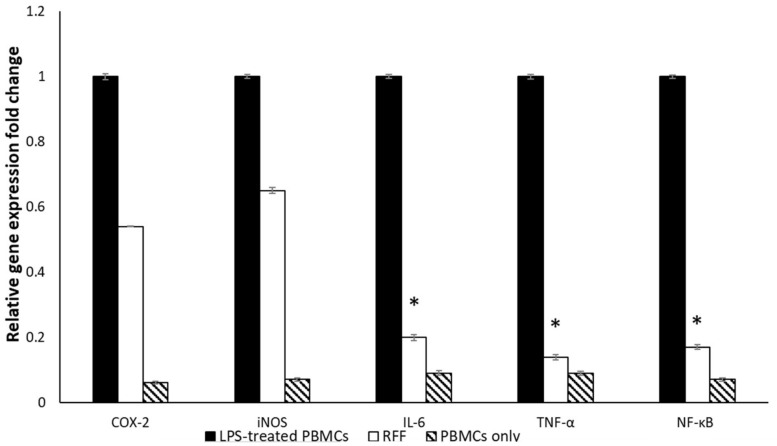
Chart presenting the impact of RRF on the relative gene expression of COX-2, iNOS, IL-6, TNF-α, and NF-κB in LPS-induced PBMCs. The artistic symbol (*) represents a significant decrease in gene expression (*p* < 0.05).

The gene expression of COX-2, iNOS, IL-6, TNF-α, and NF-κB was significantly increased in lipopolysaccharide (LPS)-induced PBMCs. Noteworthy, the treatment of LPS-induced PBMC with 0.5 IC_50_ of RRF attenuated the rise in the gene expression of cyclooxygenase-2 (COX-2) and nitric oxide synthase (iNOS). In addition, it markedly decreased (*p* < 0.05) the expression of (IL-6), tumor necrosis factor-alpha (TNF-α), and nuclear factor kappa B (NF-κB) compared to the non-treated cells, as presented in Figure 5.

### 2.4. In Vivo Activities

The effects of RRF were investigated on different biomarkers and skin histopathology following excisional wound healing. These effects shall be presented in the following subsections.

#### 2.4.1. Wound-Healing Rates

**Figure 6:**
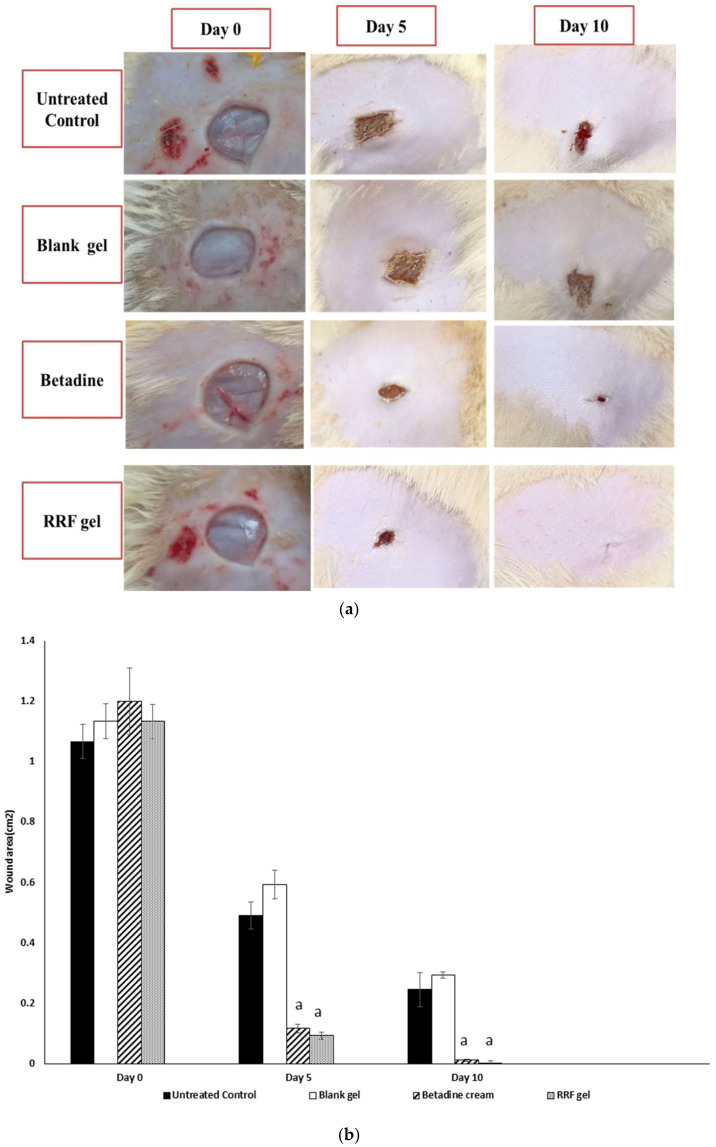
(**a**) Macroscopic visualization of the wound surface in treatment groups, Untreated control, Blank gel, Betadine, and RRF gel groups at day 0, day 5, and day 10. (**b**) Wound area in each test group (Untreated control, Blank gel, Betadine, RRF gel groups). Results were expressed as mean ± standard deviation (SD). Significant difference vs. the respective untreated control, each at *p* < 0.05.

Macroscopic healing rates of wounds of the studied group were examined on days 0, 5, and 10, in all groups, respectively (Figure 6a). Betadine and RRF gel groups demonstrated significant wound healing on day 5 (90.27, 91.76% respectively) compared to untreated control and full wound healing on day 10 (99.02, 99.70% respectively) when compared to the untreated control group (Figure 6b).

#### 2.4.2. NO Levels

The antioxidant activity of RRF was assessed by measuring NO tissue levels. The experimental findings revealed marked suppression of NO levels by betadine and RRF treatment compared to the untreated control group (55.76, 59.54%, respectively) (Table 2), *p* < 0.05.

#### 2.4.3. Inflammation Markers

The results showed that RRF treatment induced a significant downregulation of inflammatory cytokines levels IL-6 and IL-1β levels compared to the untreated control group (80, 77.11%, respectively). In addition, the Betadine-treated group showed marked anti-inflammatory activity exhibited by a marked decrease in IL-6 and IL-1*β* levels (58.55%, 69.07% respectively) in comparison to the untreated group (Table 2), *p* < 0.05.

#### 2.4.4. Gene Expression Levels of PDGF, VEGF, KGF, and Fibronectin

RRF treatment showed a marked increase in angiogenesis manifested by a pronounced up-regulation in VEGF gene expression levels (150%) compared to the untreated control group. Furthermore, the betadine group induced a considerable increase in VEGF expression levels (175%) in the comparison with the untreated control group (Table 2).

**Figure 7:**
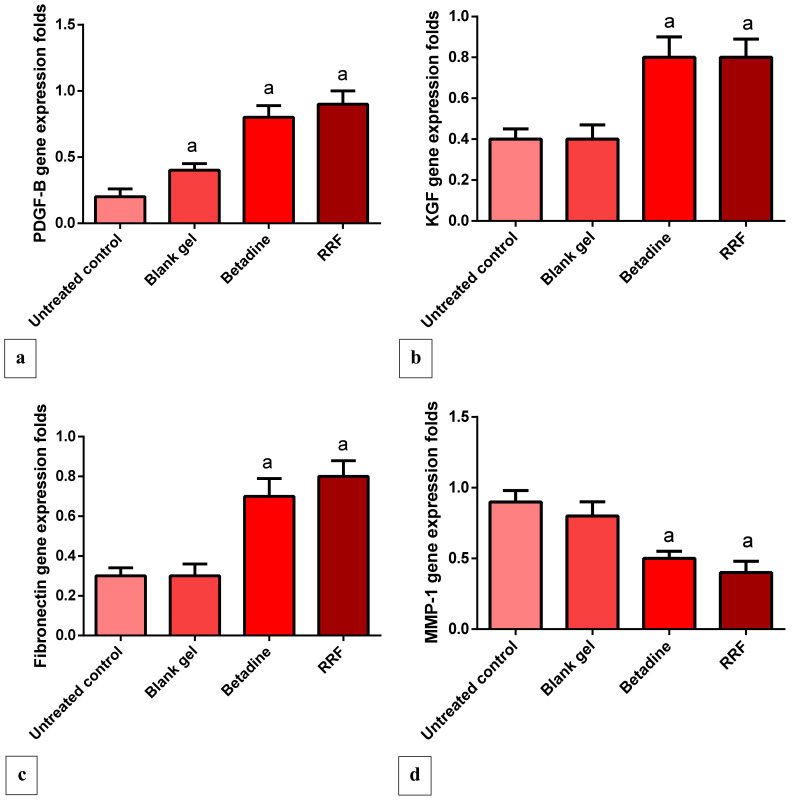
Effect of RRF treatment on (**a**) PDGF gene expression level, (**b**) KGF gene expression level, (**c**) Fibronectin gene expression level, and (**d**) MMP-1 gene expression level. Results were expressed as mean ± SD (*n* = 8/group). Significant difference vs. ^a^ respective untreated control, each at *p* < 0.05.

Compared to the untreated group, the RRF-treated group induced a pronounced elevation in platelet-derived growth factor (PDGF) expression levels (350%), and the results were comparable to the standard betadine group (Figure 7a), *p* < 0.05. In addition, when compared to the untreated group, RRF topical treatment induced a significant up-regulation in KGF gene expression levels (100%), and the results were comparable to the standard betadine group (Figure 7b).

The results showed that RRF induced a marked elevation in gene expression levels of fibronectin in comparison to the untreated control group (166%). Betadine topical treatment also up-regulated fibronectin expression levels significantly as well (133%) compared to the untreated group (Figure 7c).

#### 2.4.5. Gene Expression Levels of MMP-1 Gene

The RRF and Betadine topical treatment induced a significant suppression of MMP-1 gene expression levels (55.55, 44.4% respectively) as a comparison with the untreated control group, and these results are correlated with the degree of tissue repair and prevention of tissue damage (Figure 7d).

#### 2.4.6. Effects of RRF Topical Treatment on Immunohistochemical Staining of TGF-β

The staining intensity was scored: no staining (−), 1–25% weak staining (+), 26–50% moderate staining (++), and more than 50% strong staining (+++).

**Figure 8:**
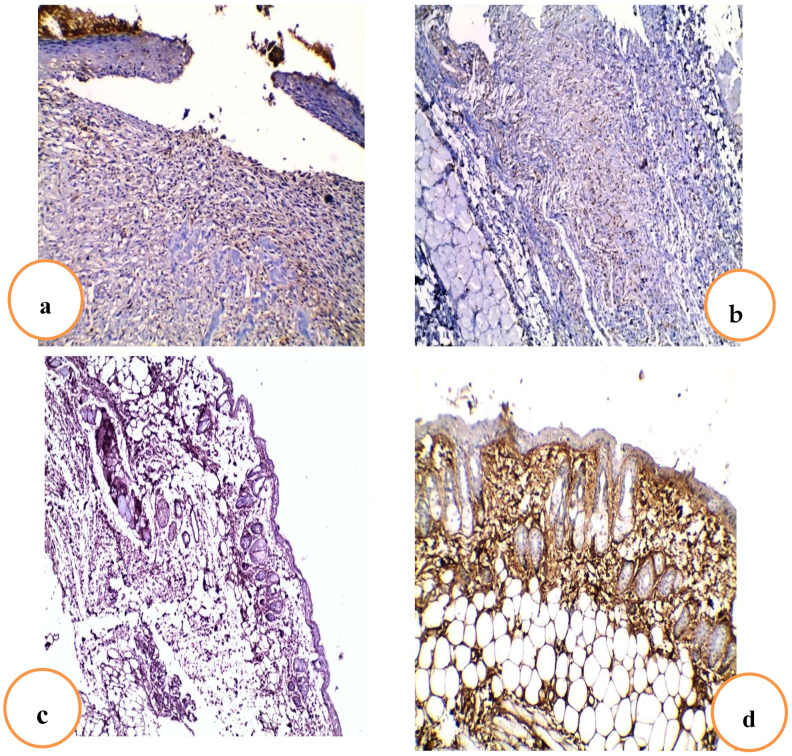
Effect of RRF treatment on TGF-*β* immunostaining in different studied groups. (**a**) Section of the skin wound of the untreated control group that showed an ulcer with underlying granulation tissue that showed weak TGF-*β* staining [×100]. (**b**) Section of the skin wound of the blank gel group that showed granulation tissue with weak TGF-*β* staining [×100]. (**c**) Section of the treated skin with the Betadine group that showed partially healed skin and regenerated thin epidermis with moderate TGF-*β* staining [×100]. (**d**) Section of treated skin with the RRF group that showed complete wound healing with strong TGF-*β* staining [×100].

The section in the skin wound of the untreated control group showed an ulcer with underlying granulation tissue that showed weak TGF-*β* staining, while section in the skin wound of the blank gel group showed granulation tissue with weak TGF-*β* staining. In addition, the section of treated skin with the Betadine group showed partially healed skin, regenerated thin epidermis with moderate TGF-*β* staining. In addition, the section of treated skin with the RRF group showed complete wound healing with strong TGF-*β* staining (Figure 8a–d).

#### 2.4.7. Histopathological Examination of Skin Tissue

**Figure 9:**
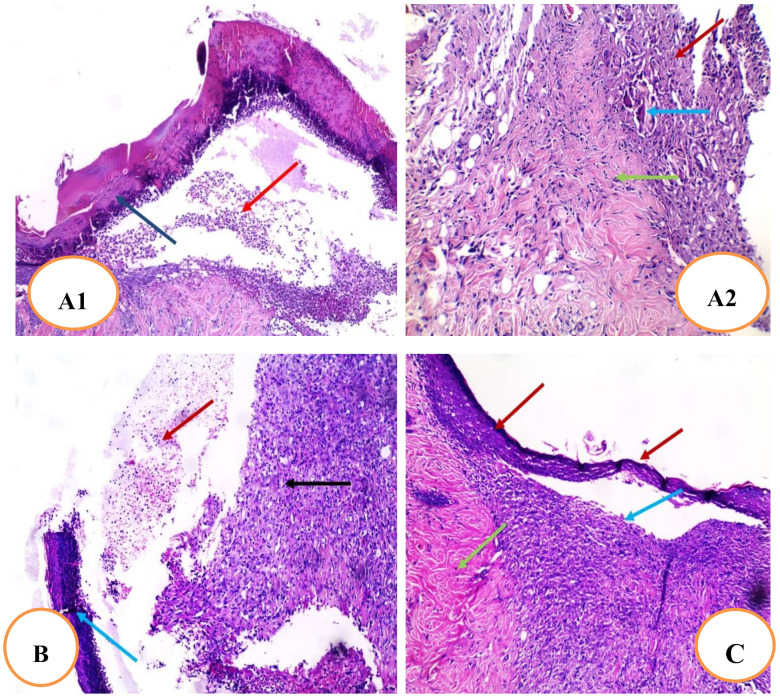

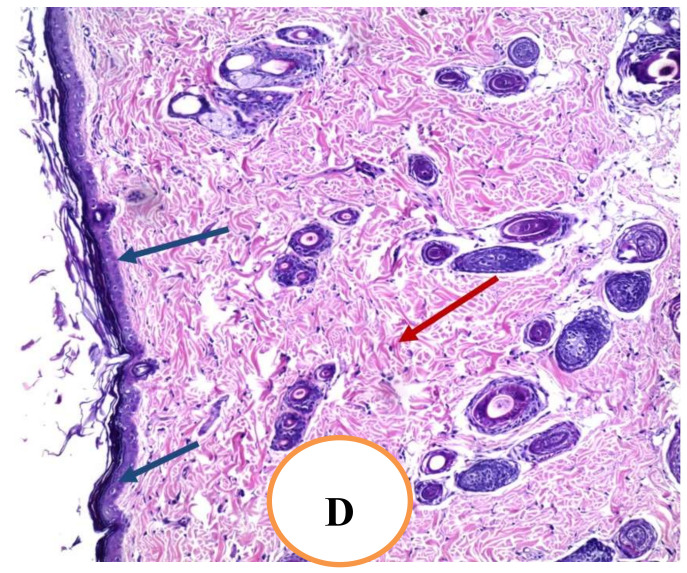
Effect of RRF treatment on histopathological examination of skin tissues of different studied groups. (**A1**) Section of the skin wound of the untreated control group showed an ulcer covered by a scab (blue arrow) filled with acute and chronic inflammatory cellular infiltrate, granulation tissue, and fibrosis (red arrow) (H&E × 100). (**A2**) Section of the skin wound of the untreated control group showed granulation tissue consisting of newly formed blood vessels surrounded by acute and chronic inflammatory cellular infiltrate mainly giant cells (blue arrow) and collagenosis and fibrosis (green arrow) (H&E × 200). (**B**) Section of the skin wound of the blank gel group showed an ulcer covered by a scab (blue arrow) filled with acute and chronic inflammatory cellular infiltrate (red arrow), granulation tissue, and fibrosis (black arrow) (H&E × 100). (**C**) Section of the treated skin with the Betadine group showed partially healed skin, the regenerated epidermis (red arrows) with underlying granulation tissue (blue arrow) surrounded by fibrosis and collagenosis (green arrow) (H&E × 100). (**D**) Section of the treated skin with the RRF gel group showed complete wound healing with continuous epidermis (blue arrows) with underlying fibrosis and collagenosis (red arrow) (H&E × 100).

The results revealed that the section in the skin wound of the untreated control group showed an ulcer covered by a scab and filled with acute and chronic inflammatory cellular infiltrate granulation tissue and fibrosis (Figure 9A1). In addition, it also showed granulation tissue consisting of newly formed blood vessels surrounded by acute and chronic inflammatory cellular infiltrate, mainly giant cells (blue arrow) and collagenosis and fibrosis (Figure 9A2). In addition, the section in the skin wound of the blank gel group showed an ulcer covered by the scab and filled with acute and chronic inflammatory cellular infiltrate granulation tissue and fibrosis (Figure 9B). Meanwhile, the section in the Betadine-treated group showed healed skin and regenerated epidermis with underlying granulation tissue surrounded by fibrosis and collagenosis (Figure 9C). As well, the section of the RRF-treated group showed complete wound healing with continuous epidermis with underlying fibrosis and collagenosis (Figure 9D).

## 3. Discussion

The metabolic profiling of the *S. officinalis* L. root extract was in agreement with the previous literature, where the plant roots are rich in polyphenols, including hydrolysable tannins, proanthocyanidins, phenolic acids, flavonoids [9,25], and triterpenoid saponins [7,10,16]. In addition, the classical column chromatography was succeeded to isolate a major flavonoid fraction, which was rich in rhoifolin detected and identified in the total crude extract. To our knowledge, rhoifolin has never been investigated before for its wound-healing effects.

The wound healing has been reported to be highly complicated following sepsis caused by a secondary bacterial infection commonly by *P. aeruginosa* and *S. aureus*. Moreover, inflammation plays a vital role in wound healing by activating inflammatory cytokines and chemokines as well as recruiting macrophages that can help with wound healing [26]. Furthermore, the pro-inflammatory cytokines are essential in the inflammatory process and attraction of neutrophils, the removal of microorganisms and pollutants from the injury site, as well as for the stimulation of metalloproteinase production. Damaged extracellular matrices (ECM) are destroyed by MMPs throughout the healing process to aid tissue restoration [27]. Prolonged inflammation, on the other hand, may promote tissue destruction, resulting in chronic wounds, as the released cytokines and proteinase may exaggerate tissue destruction [28]. In addition, inflammation causes massive destruction to the surrounding tissues, and the wound enters a pathological state that necessitates more aggressive treatment [28]. Hence, anti-inflammatory drugs are beneficial for wound treatment.

Bacteria embedded in biofilms usually exhibit enhanced resistance to antibiotics, especially if there are polymicrobial interactions. The healing of wounds, contaminated with bacteria, could be delayed as pathogenic bacteria can interfere with the wound-healing process and can lead to impaired wound repair [29]. Moreover, bacteria contaminating the wounds could elongate the phase of wound inflammation by prolonging the production of the pro-inflammatory cytokines, leading to the failure of wound healing and the wound becoming chronic. Hence, the elimination of the bacteria contaminating the wounds is an essential step for optimum wound healing [30]. Medicinal plants could be a valuable source for many phytochemicals with a significant role in fighting bacterial infections, especially in wounds [31]. Herein, RRF led to a marked decrease (*p* < 0.05) in the formation of mono-species biofilms of *P. aeruginosa* and *S. aureus* isolates at 2× MIC and 4× MIC values. In addition, RRF resulted in a substantial reduction (*p* < 0.05) in the dual-species biofilms at 4× MIC values. Cells embedded in biofilms usually require a higher concentration of the antimicrobials as the biofilm matrix, which is composed of extracellular polysaccharides, impairs the entry of these antimicrobials to the cells of biofilms [32].

PBMCs, including lymphocytes and macrophages, are induced by LPS producing many inflammatory cytokines such as IL-6 and TNF-α. Furthermore, the expression of the genes encoding COX-2 and iNOS enzymes is upregulated, resulting in excess production of prostaglandins and nitric oxide (NO) [33]. In addition, the NF-κB transcription factor, in the LPS-induced PBMCs, induces the pro-inflammatory genes to produce huge amounts of the pro-inflammatory mediators [34]. The net result of the induction of PBMCs by LPS is the overproduction of many bioactive molecules that participate in the inflammatory reaction and might result in damage to the tissues. So, inhibition of these reactions could provide a good therapeutic effect to lessen the harmful impact of inflammation, especially in wounds [35]. Thus, we assessed the immunomodulatory effect of RRF on LPS-induced PBMCs. We found that the gene expression upregulation of IL-6, TNF-α, and NF-κB significantly decreased (*p* < 0.05) after treatment of the LPS-induced PBMC with RRF when compared to the LPS-induced PBMCs before treatment. This finding suggests that rhoifolin could be beneficial in inflammatory conditions such as wounds.

Skin wounding stimulated a marked increase in IL-6 and IL-1β levels, which were strongly suppressed by RRF topical treatment, and these results are in line with previous reports [36,37,38]. The anti-inflammatory and antioxidative effects of rhoifolin in the CFA-induced arthritis model are mediated by the NF-κB pathway, according to Peng et al. [36].

It has been found that increased levels of pro-inflammatory cytokines are linked to slowed wound healing [27]. This explains why rhoifolin has a beneficial wound-healing impact by inhibiting inflammatory cytokines, which prevents extended inflammation and thus hinders wound healing. These findings indicated that rhoifolin has anti-inflammatory properties that promote the healing process.

Furthermore, the activated macrophages act in response to pathogen invasion by releasing pro-inflammatory cytokines and inflammatory mediators such as nitric oxide. [39]. In our work, RRF treatment significantly reduced NO levels, which were higher in the blank gel and control group, and these results were consistent with Yan et al. [38]. It has been confirmed that rhoifolin could significantly alleviate the IL-1β-induced up-regulation of iNOS and COX-2. The findings suggested that rhoifolin, by reducing NO generation, could be an effective antioxidant and anti-inflammatory drug. The presence of phenols may inhibit NO generation in macrophages, which is confirmed by many studies that have shown that phenols can serve as anti-inflammatory and antioxidant agents and play a major role in oxidative stress and inflammation [28,39,40,41].

In the current study, PDGF, VEGF, KGF, fibronectin, and MMP-1 were evaluated independently. Hematoxylin and eosin (H&E) staining of the wound area and immune-staining of TGF-β were also examined. The vascular endothelial growth factor is employed in the production of granulation tissue, resulting in increased angiogenesis and wound healing [42]. The RRF induced a marked up-regulation of VEGF expression, and the results are in agreement with Eldahshan et al. [43]. The resurfacing of a wound with new epithelium is known as re-epithelialization. For optimal wound healing, the cellular and molecular processes involved in the initiation, maintenance, and completion of epithelialization are critical. Growth factors, cytokines, matrix metalloproteinases, cellular receptors, and ECM components are among the modulators involved [44].

The keratinocyte growth factor is a member of the fibroblast growth factor family with a molecular weight of 28 kd that induces the proliferation of vast numbers of epithelial cells, including keratinocytes within the epidermis and dermis, and thus promotes wound healing. The RRF treatment induced a marked increase in KGF expression levels, promoting wound healing compared to the control and blank gel group. Additionally, the observed in vivo wound healing can be linked to the flavonoid nature of rhoifolin that accelerates wound healing [40]. Fibronectin is an adhesive molecule that is important in all stages of wound healing, especially ECM production and re-epithelialization [45,46,47].

Several recent studies have revealed that topical fibronectin administration can aid in the healing of chronic skin and corneal ulcers. It also aids wound healing by promoting re-epithelialization, granulation tissue, and the restoration of adequate connective tissue strength by contributing to hemostasis, assisting in infection control, and promoting re-epithelialization, granulation tissue, and the restoration of adequate connective tissue strength [45,46,47,48,49]. The RRF treatment induced a significant increase in fibronectin expression levels compared to the control and blank gel group.

The exaggerated and dysregulated protease activity may cause the degradation of adhesion proteins, preventing the cell adhesion necessary for normal wound healing [50]. The wound is associated with excessive MMP-1 expression and the degradation of ECM proteins, which is strongly attenuated by rhoifolin treatment corresponding also to the effectiveness of the wound-healing process. Herein, Betadine and RRF gel-treated groups showed greater fibronectin synthesis and diminished MMP-1 gene expression levels compared to blank and control groups, and these effects are correlated with the strength of epithelial regeneration.

PDGF is essential for wound healing at all stages. PDGF is released by degranulating platelets and found in wound fluid after an injury [3,51], which in turn stimulates chemotaxis to the wound site [28]. In addition, it promotes the production of growth factors such as TGF-*β* by macrophages. In the present study, RRF treatment induced a marked up-regulation in PDGF expression levels compared to the control and blank gel group.

Transforming growth factor-*β* signaling is essential for re-epithelialization, inflammation, angiogenesis, and granulation tissue formation during wound healing [46]. It enhances the wound-healing rate and helps avoid scarring [27]. In this investigation, it was found that Betadine and RRF gel groups significantly increased TGF-*β* immunostaining higher than control and blank gel groups. TGF*-*β is also involved in collagen production during the matrix formation and remodeling stage of wound healing. It is also a powerful inhibitor of MMP-1, MMP-3, and MMP-9, as well as a promoter of tissue inhibitor of metalloproteinase TIMP-1 production, preventing collagen degradation [3,52]. This could indicate that rhoifolin promoted wound healing by increasing TGF-*β* expression in wound tissues, which inhibited MMP-1 expression.

## 4. Materials and Methods

### 4.1. Animals and Ethical Approval

White albino rats (male, 180–210 g, 8 weeks old) were procured from the animal house at Cairo University’s College of Veterinary Medicine (Cairo, Egypt). All rats were kept in pathogen-free environments with a 12 h light/dark cycle and a constant temperature of 25 ± 2 °C as well as free access to a standard pellet diet (El-Nasr, Abuzabal, Cairo, Egypt) and filtered water (the standard pellet composition is 60% corn flour for starch, 20% fish meal for protein, 10% wheat flour or bran flour for fibers, 7% oil seed cake, 2% bone meal, and 1% salt for 1 kg). Before being used in research, all rats were given a one-week acclimatization period. The experiment was carried out following the criteria for the care and use of laboratory animals, which were authorized by the Research Ethical Committee (Faculty of Pharmacy, Tanta University, Egypt, Approval No (PO 00103).

### 4.2. Plant Material

*Sanguisorba officinalis* radices were acquired from Bozhou Swanf Commercial and Trade Co., Ltd., China. Dr. Esraa Ammar, Plant Ecology Assistant Professor, Tanta University, Faculty of Science, confirmed the plant’s identity. A voucher specimen (PGA-SO-126-W) was preserved in the herbarium of the Department of Pharmacognosy, Tanta Pharmacy.

### 4.3. LC-ESI-MS/MS and Metabolomics Analyses

#### 4.3.1. Sample Preparation and Injection

Then, the ethanol root extract of *S. officinalis* was subjected to LC-ESI-MS/MS analysis following the methods previously reported by Mohammed et al. [53] and Attallah et al. [54]. Briefly, the sample residue (50 mg) was reconstituted in 1 mL of water/methanol/acetonitrile (50:25:25, *v*/*v*%) by vortexing and ultra-sonication for 2 min and 10 min, respectively. Afterward, 10 µL of a 2.5 µg/mL solution were injected in comparison with a blank sample consisting of reconstitution solvent.

#### 4.3.2. Acquisition Method and Analytical Parameters

Analysis was conducted in a Proteomics and Metabolomics Unit, Children’s Cancer Hospital (57357), Basic Research Department, Cairo, Egypt. In-line filter disks (0.5 µm × 3.0 mm, Phenomenex^®^, Torrance, CA, USA) and X select HSS T3 (2.5 µm, 2.1 × 150 mm, Waters^®^, Milford, MA, USA, 40 °C) were used as a pre-column and analytical column, respectively. The mobile phases consisted of buffer A (5 mM ammonium formate buffer pH 3 containing 1% methanol), buffer B (5 mM ammonium formate buffer pH 8 containing 1% methanol), and buffer C (100% acetonitrile). The flow rate was adjusted at 0.3 mL/min. The liquid chromatography (ExionLC -High flow LC-, Sciex^®^, Framingham, MA, USA) was programmed to use a mobile phase composition of buffer A and C, in positive mode, while it was programmed to use a mobile phase composition of buffer B and C in negative mode. The mobile phase composition started with 90 (A or B): 10 (C) for the first 20 min, which was inversed from 21 to 25 min, and finally returned back for the last 3 min until the end of the protocol at the 28 min. In addition, the instrument was coupled with Triple TOF 5600+ (Sciex^®^) for IDA acquisition and Analyst TF 1.7.1 (Sciex^®^) for LC-Triple TOF control.

#### 4.3.3. Data Processing

MasterView was used for feature (peaks) extraction from total ion chromatogram (TIC) based on a signal-to-noise ratio greater than 5 (non-targeted analysis) and intensities of the sample-to-blank of greater than 3. In addition, Reifycs Abf (Analysis Base File) Converter (Reifycs^®^, Tokyo, Japan) was applied for Wiff file conversion and MS-DIAL 4.6 (RIKEN^®^ Tokyo, Japan) for data analysis. The used ReSpect Database possessed 1573 and 2737 records for negative and positive mode, respectively. Metabolite’s annotation was conducted with the ReSpect Database and fragmentation pattern and retention times mentioned in previous reports for metabolites isolated from the investigated plant or others.

### 4.4. Isolation of Major Flavonoid Fraction

The powdered plant (900 g) was extracted with ethanol by cold maceration (3 × 5 L). To obtain a residue (67.62 g), the extract was concentrated at reduced pressure. Then, the total crude extract (60 g) was suspended in deionized water and applied to the Diaion HP-20 column. The column was initially eluted with deionized water followed by 100% MeOH. Methanol fraction (23.5 g) was subjected to further investigation and was chromatographed over VLC (silica gel 100 g, ϕ 5 × 12 cm) eluted with CH_2_Cl_2_ and then adding MeOH in 1% increments. After TLC observation using Camag UV lamp at 254 and 366 nm, fractions were divided into seven groups from SO-1 to SO-7. Isocratic CC on silica gel with (90:10) CH_2_Cl_2_:MeOH was performed on fraction SO-3 (1240 mg) followed by Sephadex LH-20 with MeOH to give a major flavonoid fraction that contained one major and two minor spots. This fraction was subjected to LC-ESI/MS to identify its flavonoids, following the protocol used by Ibrahim and El-Banna [55].

### 4.5. Topical Gel Preparation

The gel was formulated by mixing Carbopol aqueous gel and hydroxypropyl cellulose gel. Firstly, 0.5 g Carbopol dispersed in water, 7 g glycerin, and 20 g isopropyl alcohol were blended by slowly stirring using a mechanical stirrer at 25 °C, and 3.5 g of triethanolamine in water was transferred. The mixture was further filled with water up to 100 g weight. Following, the blend was stirred constantly until the formation of a clear gel. In addition, a hydroxypropyl cellulose gel (2%) was prepared. In a 1:1 ratio, carbopol and hydroxypropyl cellulose gels were combined, and 2% of RRF was applied after gentle stirring. A blank gel was also made in the same way.

### 4.6. Antibiofilm Activities

#### 4.6.1. Isolation and Identification of *P. aeruginosa* and *S. aureus* from Wounds

*P. aeruginosa* and *S. aureus* were isolated from wound swab samples from Tanta University Hospital, Tanta, Egypt. They were identified by microscopical examination and culture on blood agar. Characterization of the isolated bacteria was carried out using standard biochemical tests [56].

#### 4.6.2. Effect of RRF on Planktonic Cells

Minimum inhibitory concentrations values of RRF against the tested bacterial isolates were identified using broth microdilution as previously described [57]. In brief, bacterial isolates were grown overnight in Mueller–Hinton Broth (MHB) (Oxoid, Lenexa, KS, USA). Then, in 96-well microtitration plates, 50 µL of serially diluted RRF in MHB (two-fold dilution) was added to 50 µL of the diluted culture. This was performed in triplicate. Microtitration plates were incubated overnight at 37 °C, and the optical density (OD) at 600 nm was measured using a spectrophotometer (Shimadzu, Kyoto, Japan).

#### 4.6.3. Biofilm Formation

*P. aeruginosa* and *S. aureus* were screened for biofilm formation as previously described using crystal violet assay [58]. About 100 µL of bacterial suspension was inoculated in 96-well microtitration plates using positive control (bacterial suspension) and negative control (broth) wells in each plate. The plates were incubated for 24 h at 37 °C; then, the contents of the wells were gently removed, and the wells were rinsed with water three times for removing the planktonic bacteria. About 100 µL of methanol was added to each well for biofilm fixation. Crystal violet was used for staining the fixed bacteria for 10 min. After that, the wells were washed three times by water and left to dry in the air. Finally, the optical density at 490 nm (OD_490_) was measured using ELISA Reader (Sunrise Tecan, Austria) after using the acetic acid solution for solubilization of the dye bound to biofilm.

Based on the measured OD, the isolates were categorized into 4 classes as follows:

a—bacteria not forming biofilm (ODc < OD < 2 ODc);

b—bacteria form biofilm weakly (2 ODc < OD < 4 ODc);

c—bacteria form biofilm moderately (4 ODc < OD < 6 ODc); and

d—bacteria form biofilm strongly (6 ODc < OD).

Cut-off OD (ODc is the mean OD of the negative control plus three standard deviations.

#### 4.6.4. Effect on Mono-Species and Dual-Species Biofilms

The RRF impact on mono-species and dual-species biofilms was evaluated. In brief, bacterial colonies of strong biofilm-forming *P. aeruginosa* and *S. aureus* isolates alone and/or mixed were transferred to 5 mL of tryptic soy broth (TSB) (Oxoid, Lenexa, KS, USA) and incubated at 37 °C for 24 h in a shaking incubator (Fredericton, NB, Canada). The selection of the isolates to be mixed was based on the MIC values of RRF. Each of the *P. aeruginosa* and *S. aureus* isolates with a similar or close to RRF MIC values were mixed to form dual-species biofilm. Then, they were centrifuged, washed thrice with phosphate-buffered saline (PBS), and resuspended in TSB. Biofilms were formed by adding 100 µL of the bacterial suspensions of *P. aeruginosa* and *S. aureus* bacteria, with concentrations of 1 × 10^7^ CFU/mL, into flat-bottomed 96-well microtitration plates and overnight incubation at 37 °C. In the case of the dual-species biofilm assay, we used the bacterial suspensions with a ratio of 1:1. Positive control wells containing bacteria only and negative control wells containing TSB only were included in each microtitration plate. After incubation, the non-adherent cells were removed, and the formed biofilms were washed three times with PBS. RRF, at concentrations equal to 0.5× MIC, 1× MIC, 2× MIC, and 4× MIC values, were added to the washed biofilms, and the plates were incubated for another 24 h [59]. The bacterial viability was assessed in the formed biofilms using (colony-forming unit) CFU assay. After incubation for 24 h, the media was removed from each well and then washed with PBS. The biofilms were separated from the wells by vortexing and transferred to Eppendorf tubes. They were serially diluted using PBS and cultured onto cetrimide agar (selective media for *P. aeruginosa*) and mannitol salt agar (selective media for *S. aureus*) (Oxoid, Lenexa, KS, USA) using the drop plate assay. The plates were incubated at 37 °C for 24 h, and the colonies were counted [59].

### 4.7. Immunomodulatory Activity

#### 4.7.1. Isolation of PBMCs

Blood from healthy donors was used for the isolation of PBMCs using ficoll density gradient centrifugation. Then, PBMCs were seeded in six-well plates in Roswell Park Memorial Institute (RPMI 1640) medium supplied with heat-inactivated fetal bovine serum (10%), penicillin–streptomycin solution (1%), and 2 mM L-glutamine. They were maintained by incubation at 37 °C for 24 h in an atmosphere with 5% CO_2_ [60].

#### 4.7.2. MTT Cell Viability Assessment

The toxicity of RRF on PBMC was assessed at a concentration range of 3.125 to 400 µg/mL, using MTT viability assay as previously described [61]. The mean inhibitory concentration of RRF (IC_50_) on PBMC was calculated, and the immunomodulatory effect of RRF was evaluated on LPS-induced PBMC at 1/2 IC_50_.

#### 4.7.3. qRT-PCR

The impact of RRF on the gene expression of COX-2, iNOS, IL-6, TNF-α, and NF-κB in LPS-induced PBMC was studied [62]. In brief, after overnight culturing 2 × 10^6^ cells/mL of PBMCs in RPMI 1640 medium, they were treated with 100 μL LPS (20 ng/mL), in the presence and absence of 0.5 IC_50_ of RRF, for another 24 h. The effect on the gene expressions of COX-2, iNOS, IL-6, TNF-α, and NF-κB was assessed by qRT-PCR (used primers are presented in Table S3) in the LPS-treated cells and the LPS -untreated cells (untreated control). A RNeasy mini kit (Qiagen, Hilden, Germany) was used for the extraction of the total RNA from PBMCs. Then, RNA was converted into complementary DNA (cDNA) using the SensiFAST™ kit (Bioline, London, UK). The utilized housekeeping gene was GAPDH, and the utilized RT-PCR master mix was SensiFAST™ SYBR green (Bioline, London, UK). The 2^−ΔΔCT^ method was utilized for the calculation of the gene expression fold change [63].

### 4.8. Wound Model and Experimental Groups

A total of 32 rats were randomly divided into four groups each with 8 rats; the Untreated control group; Blank gel (Vehicle) group; Betadine^®^ ointment (10%) (Mundi pharma, Standard drug) group, and RRF gel (2%) group. Animals were anesthetized with diethyl ether, and then, a small area on the back of each rat was shaved carefully. In the dorsal skin, full-thickness excisional skin wounds were generated according to the method described previously [64]. For ten days, the gel was administered topically on the wound’s surface [28].

#### 4.8.1. Macroscopic Wound Healing

The wounding day was classified as day 0, and the wound-healing process was observed from day 0 to day 10 after the wound. On days 0, 5, and 10, wound images were taken with a digital camera. The wound area was determined by the method described previously [65], and the percentage of the wound healing is determined according to the following formula described previously [27]

(1) $$Percentage\;of\;wound\;healing=\frac{wound\;area\;at\;day\;0-wound\;area\;at\;n\;th\;day}{wound\;area\;at\;day\;0}\times 100$$

where *n* represented day 5 or day 10.

#### 4.8.2. Determination of NO Level

The content of NO in the skin was determined using the reported method [66]. The Griess reagent can be used to detect nitrite and nitrate, which provide a reliable and quantitative estimate of NO output. A UV-PC 1601 double-beam spectrophotometer was used to measure the absorbance of each sample at 540 nm (Shimadzu, Kyoto, Japan). The standard curve was prepared by dissolving sodium nitrite in distilled water to produce a 1M solution; then, several serial dilutions were made to prepare 1 µM solution, which served as a stock solution from which the following dilutions were made to construct the nitrite standard curve (10, 20, 30, 40, 50, and 60 nM). A half mL of each dilution was mixed with 0.5 mL Griess reagent. Samples were allowed to incubate at 37 °C for 30 min, and the absorbance of samples was measured at 540 nm using a double-beam spectrophotometer. Extrapolation from the sodium nitrite standard curve was used to determine the levels of NO in each sample (Figure S5).

#### 4.8.3. Enzyme-Linked Immunosorbent Assay for IL-6 and IL-1*β* Levels

The level of inflammatory mediators IL-1*β* and IL-6 in skin tissues was assessed according to the manufacturer protocol. The levels of IL-1β and IL-6 were assessed according to the method described in commercial ELISA kits (Abcam Co. Waltham, MA, USA; Sun Red biotechnology Co., Shanghai, China) respectively, and they were expressed as pg/mg protein. To prepare the rat IL-6 standard curve, the rat IL-6 original standard was reconstituted to 640 pg/mL with standard diluent buffer. The reconstituted solution was mixed gently and allowed to sit for 10 min to ensure complete reconstitution. From the reconstituted solution, the following dilutions were made: 20, 40, 80, 160, and 320 pg/mL. Standard solutions were processed as described by manufacturer protocol to construct the IL-6 standard curve. In addition, the rat IL-1*β* original standard was reconstituted to 50,000 pg/mL with standard diluent buffer. The reconstituted solution was mixed thoroughly and left aside for 10 min to ensure complete reconstitution. From the reconstituted solution, the following dilutions were made (68.59, 205.8, 617.3, 1852, 5556, and 16,667 pg/mL). Standard solutions were processed as stated by the manufacturer’s protocol to construct the IL-1*β* standard curve. Extrapolation from standard curves was used to determine the levels of IL-6 and IL-1*β* in each sample (Figures S6 and S7).

#### 4.8.4. Quantitative Real-Time (qRT-PCR) for PDGF, KGF, VEGF, MMP-1, and Fibronectin Genes

According to the manufacturer’s procedure, the total RNA was purified from skin samples using TRIzols Reagent (15596026) (Life Technologies, Carlsbad, CA, USA). In a two-step RT-PCR experiment, 1 μg of total RNA was reverse-transcribed into single-stranded complementary DNA using the QuantiTects Reverse Transcription Kit (Qiagen, Hilden, FL, USA) and a random primer hexamer. QuantiTect Reverse Transcriptase is a hybrid of Omniscript and Sensiscript reverse transcriptases with a high affinity for RNA and the ability to produce cDNA from a wide range of RNA concentrations (10 pg to 1 μg). Unlike other kits, the QuantiTect Reverse Transcription Kit produces high quantities of cDNA templates for real-time PCR analysis independent of the location of the amplified target area on the transcript. C-DNA amplicons were amplified via Maximas SYBR Green/Fluorescein qPCR Master Mix (Thermo Fisher Scientific, Waltham, MA, USA) through specific primers (as shown by Table S4), which were prepared according to the manufacturer’s protocol.

Thermal cycling conditions were designed as follows: 10 min at 95 °C, followed by 45 cycles of 95 °C for 10 s, 60 °C for 15 s, and 72 °C for 15 s. The conditions of the melting curve analysis were 72–95 °C, increased by 1 °C s^−1^. Finally, the 2^−ΔΔCT^ method was performed to measure relative mRNA expression and normalized to *β*-actin [67].

### 4.9. Histopathological Examination of Skin Sections

The entire wound was removed for histological assessment at the end of the trial, with a margin of roughly 5 mm of surrounding unwounded skin. Skin sections were fixed in a 10% formalin solution (pH 7.4) for 24 h before being processed through a series of alcohol and xylene grades. At 65 °C, the tissues were ultimately embedded in paraffin wax. Tissue blocks were cut into 5 μm thick sections, stained with hematoxylin and eosin (HandE), and viewed under a light microscope.

### 4.10. Immunohistochemical Staining of TGF-β

The skin samples were fixed in a 10% neutral formalin solution. Then, they were bisected, immersed in paraffin, and sectioned in 5 µm thick layers after 24 h. The 5 µm thick sections were mounted on glass slides, dewaxed, rehydrated with distilled water, and stained with TGF-*β* (ABclonal Technology, Woburn, MA, USA). TGF-*β* staining was examined at a magnification of 100× in all fields of tissue slices. The presence of cytoplasmic staining was regarded as a favorable sign. The strength of the staining was graded as follows: no staining (−), 1 to 25% weak staining (+), 26 to 50% moderate staining (++), and more than 50% strong staining (+++) [68].

### 4.11. Statistical Analysis

Results were represented as mean ± SD. All calibration curves were subjected to regression analysis, and correlation coefficients were calculated. A one-way analysis of variance (ANOVA) was used to compare distinct groups, followed by a Tukey–Kramer post hoc test. The significance level was chosen at *p* < 0.05. Prism version 6 was used to conduct the statistical analysis (GraphPad Software, Inc., San Diego, CA, USA).

## 5. Conclusions

RRF isolated from *S. officinalis* L. radix was explored for its wound-healing properties for the first time to the best of our knowledge. The fraction exhibited promising antibiofilm activity against mono-species and dual-species biofilms of *P. aeruginosa* and *S. aureus* bacteria isolated from wounds. Moreover, it exhibited an immunomodulatory activity on LPS-induced PBMCs. The current investigation confirmed also that RRF has a promising wound-healing effect via up-regulating PDGF, VEGF, KGF, fibronectin, and suppressing ECM degradation through down-regulating MMP-1 expression levels. Moreover, it exerted anti-inflammatory effects, enhanced TGF-*β* immune-staining, and improved histopathological changes. Based on these findings, it is concluded that RRF may be used as a good alternative for wound healing via supporting anti-bacterial, re-epithelization, angiogenesis, and anti-inflammatory activity. Yet, more clinical and pre-clinical investigations are required to be performed on the fraction assessing the beneficial role in wound healing of human injuries.

## Figures and Tables

**Table 1:** List of tentatively identified metabolites in ethanol extract of *Sanguisorba officinalis* roots analyzed by LC-ESI-MS/MS.

No.	Peak Area (%)	Identified Metabolite	RT (min)	Molecular Formula		[M − H]^−^ *m/z*	[M + H]^+^ *m/z*	MS^2^ Fragments (*m/z*)	Ref
Phenolic acids/glycoside									
1	0.11	Syringoylmalic acid	2.7	C_13_H_14_O_9_			315.070	125.0, 153.0, 169.1, 297.1	[21]
2	0.07	Rosmarinic acid	4.1	C_18_H_16_O_8_		359.096		150.9, 169.0, 188.9, 314.9	--- *
3	0.01	Homogenentisic acid	4.46	C_8_H_8_O_4_		167.034		82.9, 109.0, 122.9, 122.9, 149.0	---
4	0.2	Syringic acid	7.21	C_9_H_10_O_5_			199.059	59.0, 65.0, 95.0, 107.0, 123.0, 125.0, 135.0, 139.0, 140.0, 152.0, 167.0	[21]
5	0.22	1-O-*β*-_D_-glucopyranosyl sinapate	14.32	C_17_H_22_O_10_			387.178	77.0, 93.0, 105.0, 119.0, 121.0, 147.0	---
6	0.21	Unknown phenolic acids/glycoside	21.61	C_16_H_24_O_7_			329.159	111.0, 129.0, 139.0, 157.0, 185.0	---
Alkaloids and related metabolites									
7	0.07	Harmaline	1.21	C_13_H_14_N_2_O			215.099	127.1, 144.0, 157.0, 183.0	---
8	0.03	3-Methyl xanthine	7.21	C_6_H_6_N_4_O_2_			167.034	60.0, 149.0, 152.0	---
Flavonoids and related metabolites									
9	0.02	Quercetin-3-_D_-xyloside	1.34	C_20_H_18_O_11_		433.038		299.9, 300.9, 366.9	---
10	0.12	Epigallocatechin	2.71	C_15_H_14_O_7_		305.063		125.0, 165.0, 179.0, 221.0, 261.0	---
11	0.07	Eriodictyol-7-*O*-glucoside	3.71	C_21_H_22_O_11_		449.110		229.0, 259.0, 269.0, 274.9, 287.0	---
12	0.81	Procyanidin B2	4.29	C_30_H_26_O_12_		577.131		125.0, 137.0, 161.0, 205.0, 245.0, 247.0, 273.0, 275.0, 287.0, 289.0, 299.0, 339.0, 381.1, 407.0, 425.0, 451.1, 559.1	---
13	0.02	Procyanidin C1	4.47	C_45_H_38_O_18_		865.208		287.0, 413.1, 425.1, 575.1, 575.1 577.1, 695.1, 713.1, 713.1, 739.1	---
14	3.3	(-)-Epicatechin	4.62	C_15_H_14_O_6_		289.071		57.0, 81.0, 83.0, 95.1, 97.0, 108.0, 123.0, 125.0, 135.0, 137.0, 139.0, 149.0, 151.0, 161.0, 162.0, 164.0, 165.0, 167.0, 175.0, 179.0, 187.0, 188.0, 202.0, 203.0, 205.0, 220.9, 221.0, 227.0, 230.0, 247.0	---
15	0.51	Catechin	4.73	C_15_H_14_O_6_			291.085	68.0 77.0, 91.0, 93.0, 105.0, 111.0, 115.0, 119.0, 123.0, 127.0, 133.0, 137.0, 137.0, 139.0, 143.0, 147.0 151.0, 161.0, 163.0, 165.0, 177.0 179.0, 189.0, 207.0, 249.0, 273.0	---
16	0.2	3,5,7-trihydroxy-4′-methoxyflavone	5.12	C_16_H_12_O_6_		298.982		79.9, 181.0, 283.9	---
17	0.003	Luteolin-6-*C*- Glucoside	5.2	C_21_H_20_O_11_		447.094		174.9, 299.9, 303.0, 315.0, 327.0, 378.9, 401.1	---
18	0.12	Procyanidin B1	5.46	C_30_H_26_O_12_		577.134		125.0, 165.0, 287.0, 289.0, 299.0, 381.1, 407.1, 425.1, 451.1	---
19	0.02	Naringenin-7-*O*- Glucoside	5.8	C_21_H_22_O_10_		433.114		123.0, 135.0, 163.0, 188.9, 237.0, 253.0, 271.0, 296.9	---
20	0.2	Isookanin-7-glucoside	5.85	C_21_H_22_O_11_		449.109		150.9, 178.9, 259.0, 269.0, 287.0	---
21	0.06	Kaempferol-3- Glucuronide	6.53	C_21_H_18_O_12_		461.072		188.9, 256.9, 313.9, 324.9, 328.0, 329.0, 392.8	---
22	0.01	Quercetin-3- Arabinoside	7.17	C_20_H_18_O_11_			435.164	273.0, 302.9, 303.0	---
23	0.03	Phlorizin	7.77	C_21_H_24_O_10_		435.129		167.0, 180.0, 271.0, 273.0	---
24	0.03	Isorhamnetin-3-*O*-glucoside	6.78	C_22_H_22_O_12_		477.142		163.0, 169.0, 313.0, 324.9, 364.8, 432.8	---
25	0.01	4,5′-dihydroxy-3-methoxy-3′-glucopyranosylstilbene	6.85	C_21_H_24_O_9_		419.099		259.0, 282.9, 287.0, 351.0	---
26	0.003	Rhoifolin (Apigenin 7-*O*-neohesperidoside)	7.24	C_27_H_30_O_14_		577.213		112.9, 356.9	---
27	0.004	Neohesperidin dihydrochalcone	8.08	C_28_H_36_O_15_		611.141		400.8, 520.8, 565.0	---
28	0.03	Kaempferol-3-*O*-α-_L_-arabinoside	8.13	C_20_H_18_O_10_		417.117		119.0, 218.9, 255.0, 280.9, 286.9, 354.9	---
29	0.01	4-deoxyphloridzin	8.29	C_21_H_24_O_9_		419.133		151.0, 257.0, 351.0	---
30	0.02	Naringenin	9.94	C_15_H_12_O_5_		271.062		93.0, 119.0, 151.0, 225.1, 253.0	---
31	0.19	4,4′-Di-*O*-methylellagic acid	10.22	C_16_H_10_O_8_			331.042	225.0, 245.0, 270.0011:54 271.0, 299.0, 300.9, 316.0	---
32	0.08	3,3′,4′,5-tetrahydroxy-7-methoxyflavone	10.23	C_16_H_12_O_7_			316.971	317.0	---
33	0.01	Apigenin	10.39	C_15_H_10_O_5_		269.043		117.0, 269.0, 269.2, 269.2	---
34	0.003	Cyanidin-3-O-(2″-O-*β*-xylopyranosyl-*β*-glucopyranoside)	12.43	C_26_H_29_O_15_			581.079	564.1	---
35	0.01	Luteolin	15.93	C_15_H_10_O_6_			287.200	137.0, 203.1, 272.1	---
36	0.02	3′-Methoxy-4′,5,7-trihydroxyflavonol	16.87	C_16_H_12_O_7_			317.056	299.2, 302.0	---
37	0.01	E-3,4,5′-Trihydroxy-3′-glucopyranosylstilbene	19.85	C_20_H_22_O_9_		405.171		390.1	---
38	0.04	3,5,7-trihydroxy-4′-methoxyflavone	20.11	C_16_H_12_O_6_			301.141	161.0, 285.0	---
Triterpenoids									
39	0.1	3-Oxo-15α, 19α-dihydroxyurs-12-en-28-oic acid or 3-oxo-7*β*, 19α-dihydroxyurs-12-en-28-oic acid	11.29	C_30_H_46_O_5_		485.328		354.9, 372.9, 405.3, 423.3, 455.3	[17]
40	0.02	Ziyuglycoside I	11.35	C_41_H_66_O_13_		765.481		585.3, 601.4, 603.3, 604.3	[8]
41	0.1	Unknown triterpenoid	11.96	C_31_H_50_O_8_		549.339		421.3, 501.7, 503.3	
42	0.11	Lup-12-en-15*α*,19*β*-diol-3,11-dioxo-28-oic acid	13.41	C_30_H_44_O_6_			501.319	231.1, 341.2, 437.3, 455.3, 465.3, 483.3	[11]
43	0.3	Euscaphic acid or Arjunic acid	13.96	C_30_H_48_O_5_		488.347		424.3, 487.3, 488.3	[16]
44	0.25	3-Oxo-23-hydroxyurs-12-en-28-oic acid	14.22	C_30_H_46_O_4_			471.348	213.1, 285.2, 407.3, 425.3, 453.3	[17]
45	0.07	Unknown	15.81	C_30_H_42_O_5_			483.310	185.1, 213.1, 233.1, 419.2, 465.2	
46	0.05	Sanguisorbigenin	18.88	C_30_H_46_O_3_			455.351	187.1, 189.1, 191.1, 201.1, 409.3, 437.3	[9]
47	0.11	18,19-*Seco*,1*β*-hydroxyl-3,19-dioxo-urs-11,13(18)-dien-28-oic acid	18.98	C_30_H_44_O_5_			485.326	187.1, 205.1, 235.1, 367.2, 421.3, 439.3, 467.3	[17]
48	0.54	Unknown	19.74	C_30_H_44_O_4_			469.330	147.1, 283.2, 351.2, 405.3, 423.3	
49	0.23	Fupenzic acid	20.35	C_30_H_44_O_3_			453.337	119.1, 133.1, 145.1, 173.1, 175.1, 177.1, 205.1, 259.1, 389.3	[17]
50	0.02	Ursolic acid	22.3	C_30_H_48_O_3_		455.355		180.9, 248.9, 250.9, 318.9, 409.2	[17]
Fatty acids									
51	0.32	Linoleic acid	21.92	C_18_H_32_O_2_		279.234		210.9	---
52	0.7	Glyceryl palmitate	23.29	C_19_H_38_O_4_			331.286	57.0, 71.0, 85.0, 95.0, 109.0, 123.1, 239.2, 313.2	---
53	0.07	Glyceryl 2-linolenate	23.31	C_21_H_36_O_4_			353.263		---
54	0.06	Oleic acid	24.19	C_18_H_34_O_2_		281.251		213.2, 280.3	---
Others									
55	0.01	_D_-Carnitine	1.2	C_7_H_15_NO_3_			162.112	55.0, 59.0, 73.9, 103.0, 127.0	---
56	0.33	7-(α-_D_-Glucopyranosyloxy)-2,3,4,5,6-pentahydroxyheptanoic acid	1.24	C_13_H_24_O_13_		387.114		89.0, 161.0, 179.0, 251.0, 258.9, 263.0, 323.1, 341.1	---
57	0.002	Unknown thioglycoside	6.16	C_12_H_23_NO_10_S_3_		436.088		304.0, 388.0	---
58	1.1	Zingiberoside A	9.51	C_22_H_38_O_12_	7.6	493.225		89.0, 131.0, 149.0, 179.0, 191.0, 221.0, 251.0, 288.9, 311.0, 315.1, 356.9, 430.8, 447.2	---
59	0.2	Decanoylsucrose	10.35	C_22_H_40_O_12_		495.243		99.0, 119.0, 131.0, 317.1, 449.2	---
60	0.08	γ-Terpinene	10.76	C_10_H_16_			137.132	67.0, 77.0, 110.0, 122.0	---
61	0.03	Esculin	14.75	C_15_H_16_O_9_		339.196		189.0, 255.2, 270.9, 324.1	---
62	0.12	Unknown	16.87	C_18_H_14_O_3_			279.102	149.0, 190.0, 205.1, 233.0, 261.0	
63	1.44	Unknown	20.86	C_19_H_18_O_3_			295.133	178.0, 191.0, 192.0, 206.1, 207.0, 207.1, 219.0, 221.1, 235.0 249.1, 252.0, 262.1, 265.0, 266.0, 277.1, 280.0	

*: identified by library.

**Table 2:** Effects of RFF treatment on VEGF gene expression level, IL-6 level, IL-1*β* level, and NO content in skin wound model in rats.

	VEGF Gene Expression Folds	IL-6 Level (pg/mg Tissue)	IL-1*β* Level (pg/mg Tissue)	Skin NO Content (nmol/g Tissue)
Untreated control	0.4 ± 0.06	350.6 ± 8.65	442.3 ± 12.3	45.48 ± 3.65
Control vehicle	0.5 ± 0.09	356.3 ± 9.63	440 ± 10.9	48.53 ± 4.5
Betadine	1.1 ± 0.13 ^a^	145.3 ± 5.6 ^a^	136.8 ± 7.8 ^a^	20.12 ± 2.85 ^a^
Rhoifolin rich fraction (RRF)	1 ± 0.07 ^a^	70.1 ± 6.7^a^	101.2 ± 8.12 ^a^	18.4 ± 2.12 ^a^

Results were expressed as mean ± SD (*n* = 8/group). Significant difference vs. ^a^ respective control, each at *p* ˂ 0.05.

## Data Availability

Data is contained within the article and supplementary material.

## Supplementary Materials

The following are available online at https://www.mdpi.com/article/10.3390/ph15020178/s1, Figure S1: TIC of LC-ESI-MS/MS analysis of *S. officinalis* roots ethanol extract in negative ionization mode, Figure S2: TIC of LC-ESI-MS/MS analysis of *S. officinalis* roots ethanol extract in positive ionization mode, Figure S3: Total ion chromatogram (TIC) of rhoifolin rich fraction (RRF) isolated from *S. officinalis* roots ethanol extract, Figure S4: ESI/MS of identified compounds in rhoifolin rich fraction (RRF). The compounds were tentatively identified as neohesperidin dihydrochalcone (1), apigenin 7-*O*-neohesperidoside (Rhoifolin) (2), and isookanin-7-glucoside (flavanomarein) (3), Figure S5: NO standard curve, Figure S6: IL-6 standard curve, Figure S7: IL-1*β* standard curve. Table S1. MIC values of RRF against the tested *P. aeruginosa* isolates and the level of their biofilm-forming ability. Table S2. MIC values of RRF against the tested *S. aureus* isolates and the level of their biofilm-forming ability. Table S3. Sequences of the utilized primers (in vitro study), Table S4. Forward and reverse primer sequences used in quantitative RT-PCR (in vivo study).

## References

[1] Tanrıverdi S.T., Suat B., Azizoğlu E., Köse F.A. (2018). In-vitro evaluation of dexpanthenol-loaded nanofiber mats for wound healing. Trop. J. Pharm. Res..

[2] Benbow M. (2011). Wound care: Ensuring a holistic and collaborative assessment. Br. J. Community Nurs..

[3] Barrientos S., Stojadinovic O., Golinko M.S., Brem H., Tomic-Canic M. (2008). Growth factors and cytokines in wound healing. Wound Repair Regen..

[4] Siafaka P.I., Zisi A.P., Exindari M.K., Karantas I.D., Bikiaris D.N. (2016). Porous dressings of modified chitosan with poly (2-hydroxyethyl acrylate) for topical wound delivery of levofloxacin. Carbohydr. Polym..

[5] Yadav M.K., Chae S.-W., Go Y.Y., Im G.J., Song J.-J. (2017). In vitro multi-species biofilms of methicillin-resistant Staphylococcus aureus and Pseudomonas aeruginosa and their host interaction during in vivo colonization of an otitis media rat model. Front. Cell. Infect. Microbiol..

[6] Chen J.-f., Tan L., Ju F., Kuang Q.-x., Yang T.-l., Deng F., Gu Y.-c., Jiang L.-s., Deng Y., Guo D.-l. (2020). Phenolic glycosides from Sanguisorba officinalis and their anti-inflammatory effects. Nat. Prod. Res..

[7] Jang E., Inn K.S., Jang Y.P., Lee K.T., Lee J.H. (2018). Phytotherapeutic Activities of Sanguisorba officinalis and its Chemical Constituents: A review. Am. J. Chin. Med..

[8] Kim Y.H., Chung C.B., Kim J.G., Ko K.I., Park S.H., Kim J.H., Eom S.Y., Kim Y.S., Hwang Y.I., Kim K.H. (2008). Anti-wrinkle activity of ziyuglycoside I isolated from a Sanguisorba officinalis root extract and its application as a cosmeceutical ingredient. Biosci. Biotechnol. Biochem..

[9] Lachowicz S., Oszmiański J., Rapak A., Ochmian I. (2020). Profile and Content of Phenolic Compounds in Leaves, Flowers, Roots, and Stalks of Sanguisorba officinalis L. Determined with the LC-DAD-ESI-QTOF-MS/MS Analysis and Their in vitro Antioxidant, Antidiabetic, Antiproliferative Potency. Pharmaceuticals.

[10] Yoshida H., Yamazaki K., Komiya A., Aoki M., Kasamatsu S., Murata T., Sayo T., Cilek M.Z., Okada Y., Takahashi Y. (2019). Inhibitory effects of Sanguisorba officinalis root extract on HYBID (KIAA1199)-mediated hyaluronan degradation and skin wrinkling. Int. J. Cosmet. Sci..

[11] Zhang F., Fu T.-J., Peng S.-L., Liu Z.-R., Ding L.-S. (2005). Two New Triterpenoids from the Roots of Sanguisorba officinalis L.. J. Integr. Plant Biol..

[12] Guo D.-L., Chen J.-F., Tan L., Jin M.-Y., Ju F., Cao Z.-X., Deng F., Wang L.-N., Gu Y.-C., Deng Y. (2019). Terpene glycosides from Sanguisorba officinalis and their anti-inflammatory effects. Molecules.

[13] Li W., Yang C.-j., Wang L.-q., Wu J., Dai C., Yuan Y.-m., Li G.Q., Yao M.-c. (2019). A tannin compound from Sanguisorba officinalis blocks Wnt/β-catenin signaling pathway and induces apoptosis of colorectal cancer cells. Chin. Med..

[14] Su X.D., Ali I., Arooj M., Koh Y.S., Yang S.Y., Kim Y.H. (2018). Chemical constituents from Sanguisorba officinalis L. and their inhibitory effects on LPS-stimulated pro-inflammatory cytokine production in bone marrow-derived dendritic cells. Arch. Pharmacal Res..

[15] Zhao Z., He X., Zhang Q., Wei X., Huang L., Fang J.C., Wang X., Zhao M., Bai Y., Zheng X. (2017). Traditional Uses, Chemical Constituents and Biological Activities of Plants from the Genus Sanguisorba L.. Am. J. Chin. Med..

[16] Kim S., Oh S., Noh H.B., Ji S., Lee S.H., Koo J.M., Choi C.W., Jhun H.P. (2018). In Vitro Antioxidant and Anti-Propionibacterium acnes Activities of Cold Water, Hot Water, and Methanol Extracts, and Their Respective Ethyl Acetate Fractions, from Sanguisorba officinalis L. Roots. Molecules.

[17] Wang R., Jin M., Jin C., Sun J., Ye C., Zong T., Chen G., Zhou W., Li G. (2019). Three new ursane-type triterpenoids from the roots of Sanguisorba officinalis L. and their cytotoxic activity. Phytochem. Lett..

[18] Wang L., Li H., Shen X., Zeng J., Yue L., Lin J., Yang J., Zou W., Li Y., Qin D. (2020). Elucidation of the molecular mechanism of Sanguisorba Officinalis L. against leukopenia based on network pharmacology. Biomed. Pharmacother..

[19] Han J.H., Kim M., Choi H.-J., Jin J.S., Lee S.-O., Bae S.-J., Ryu D., Ha K.-T. (2021). The Oral Administration of Sanguisorba officinalis Extract Improves Physical Performance through LDHA Modulation. Molecules.

[20] Zhang W., Peng C., Shen X., Yuan Y., Zhang W., Yang C., Yao M. (2021). A Bioactive Compound from Sanguisorba officinalis L. Inhibits Cell Proliferation and Induces Cell Death in 5-Fluorouracil-Sensitive/Resistant Colorectal Cancer Cells. Molecules.

[21] Elshamy A.I., Farrag A.R.H., Ayoub I.M., Mahdy K.A., Taher R.F., Gendy A.E.-N.G.E., Mohamed T.A., Al-Rejaie S.S., Ei-Amier Y.A., Abd-Eigawad A.M. (2020). UPLC-qTOF-MS Phytochemical Profile and Antiulcer Potential of Cyperus conglomeratus Rottb. Alcoholic Extract. Molecules.

[22] Brinza I., Abd-Alkhalek A.M., El-Raey M.A., Boiangiu R.S., Eldahshan O.A., Hritcu L. (2020). Ameliorative effects of rhoifolin in scopolamine-induced amnesic zebrafish (Danio rerio) model. Antioxidants.

[23] Wang J., Hui Q., Qin H., Zhu W. (1992). Studies on chemical constituents of Bidens bipinnata. Chin. Tradit. Herb. Drugs.

[24] She G., Wang S., Liu B. (2011). Dihydrochalcone glycosides from Oxytropis myriophylla. Chem. Cent. J..

[25] Gawron-Gzella A., Witkowska-Banaszczak E., Bylka W., Dudek-Makuch M., Odwrot A., Skrodzka N. (2016). Chemical composition, antioxidant and antimicrobial activities of Sanguisorba officinalis L. extracts. Pharm. Chem. J..

[26] Shi G.-b., Wang B., Wu Q., Wang T.-c., Wang C.-l., Sun X.-h., Zong W.-t., Yan M., Zhao Q.-c., Chen Y.-f. (2014). Evaluation of the wound-healing activity and anti-inflammatory activity of aqueous extracts from Acorus calamus L.. Pak. J. Pharm. Sci..

[27] Alsenani F., Ashour A.M., Alzubaidi M.A., Azmy A.F., Hetta M.H., Abu-Baih D.H., Elrehany M.A., Zayed A., Sayed A.M., Abdelmohsen U.R. (2021). Wound Healing Metabolites from Peters’ Elephant-Nose Fish Oil: An In Vivo Investigation Supported by In Vitro and In Silico Studies. Mar. Drugs.

[28] Okur M.E., Karadağ A.E., Üstündağ Okur N., Özhan Y., Sipahi H., Ayla Ş., Daylan B., Demirci B., Demirci F. (2020). In vivo wound healing and in vitro anti-inflammatory activity evaluation of Phlomis russeliana extract gel formulations. Molecules.

[29] Mihai M.M., Preda M., Lungu I., Gestal M.C., Popa M.I., Holban A.M. (2018). Nanocoatings for chronic wound repair—modulation of microbial colonization and biofilm formation. Int. J. Mol. Sci..

[30] Brown M.S., Ashley B., Koh A. (2018). Wearable technology for chronic wound monitoring: Current dressings, advancements, and future prospects. Front. Bioeng. Biotechnol..

[31] Kebede T., Gadisa E., Tufa A. (2021). Antimicrobial activities evaluation and phytochemical screening of some selected medicinal plants: A possible alternative in the treatment of multidrug-resistant microbes. PLoS ONE.

[32] Limoli D.H., Jones C.J., Wozniak D.J. (2015). Bacterial extracellular polysaccharides in biofilm formation and function. Microbiol. Spectr..

[33] Kany S., Vollrath J.T., Relja B. (2019). Cytokines in inflammatory disease. Int. J. Mol. Sci..

[34] Bayraktar R., Bertilaccio M.T.S., Calin G.A. (2019). The interaction between two worlds: MicroRNAs and Toll-like receptors. Front. Immunol..

[35] Cao Y., Chen J., Ren G., Zhang Y., Tan X., Yang L. (2019). Punicalagin prevents inflammation in LPS-induced RAW264. 7 macrophages by inhibiting FoxO3a/autophagy signaling pathway. Nutrients.

[36] Peng S., Hu C., Liu X., Lei L., He G., Xiong C., Wu W. (2020). Rhoifolin regulates oxidative stress and proinflammatory cytokine levels in Freund’s adjuvant-induced rheumatoid arthritis via inhibition of NF-κB. Braz. J. Med. Biol. Res..

[37] Fang J., Cao Z., Song X., Zhang X., Mai B., Wen T., Lin J., Chen J., Chi Y., Su T. (2020). Rhoifolin Alleviates Inflammation of Acute Inflammation Animal Models and LPS-Induced RAW264. 7 Cells via IKKβ/NF-Κb Signaling Pathway. Inflammation.

[38] Yan J., Ni B., Sheng G., Zhang Y., Xiao Y., Ma Y., Li H., Wu H., Tu C. (2021). Rhoifolin Ameliorates Osteoarthritis via Regulating Autophagy. Front. Pharmacol..

[39] Murthuza S., Manjunatha B. (2018). In vitro and in vivo evaluation of anti-inflammatory potency of Mesua ferrea, Saraca asoca, Viscum album & Anthocephalus cadamba in murine macrophages raw 264.7 cell lines and Wistar albino rats. Beni-Suef Univ. J. Basic Appl. Sci..

[40] Okur M.E., Karantas I.D., Şenyiğit Z., Okur N.Ü., Siafaka P.I. (2020). Recent trends on wound management: New therapeutic choices based on polymeric carriers. Asian J. Pharm. Sci..

[41] Eldahshan O.A., Azab S.S. (2012). Anti-inflammatory effect of apigenin-7-neohesperidoside (rhoifolin) in carrageenin-induced rat oedema model. J. Appl. Pharm. Sci..

[42] Ayla Ş., Günal M.Y., Sayın Şakul A., Biçeroğlu Ö., Özdemir E.M., Okur M.E., Çiçek-Polat D., Üstündağ-Okur N., Bilgiç B.E. (2017). Effects of Prunus spinosa L. fruits on experimental wound healing. Medeni. Med. J..

[43] Eldahshan O.A. (2013). Rhoifolin; a potent antiproliferative effect on cancer cell lines. J. Pharm. Res. Int..

[44] Rousselle P., Montmasson M., Garnier C. (2019). Extracellular matrix contribution to skin wound re-epithelialization. Matrix Biol..

[45] Lenselink E.A. (2015). Role of fibronectin in normal wound healing. Int. Wound J..

[46] Rousselle P., Braye F., Dayan G. (2019). Re-epithelialization of adult skin wounds: Cellular mechanisms and therapeutic strategies. Adv. Drug Deliv. Rev..

[47] Johnson M.B., Pang B., Gardner D.J., Niknam-Benia S., Soundarajan V., Bramos A., Perrault D.P., Banks K., Lee G.K., Baker R.Y. (2017). Topical fibronectin improves wound healing of irradiated skin. Sci. Rep..

[48] Chantre C.O., Campbell P.H., Golecki H.M., Buganza A.T., Capulli A.K., Deravi L.F., Dauth S., Sheehy S.P., Paten J.A., Gledhill K. (2018). Production-scale fibronectin nanofibers promote wound closure and tissue repair in a dermal mouse model. Biomaterials.

[49] Patten J., Wang K. (2021). Fibronectin in development and wound healing. Adv. Drug Deliv. Rev..

[50] Reiss M.J., Han Y.-P., Garcia E., Goldberg M., Yu H., Garner W.L. (2010). Matrix metalloproteinase-9 delays wound healing in a murine wound model. Surgery.

[51] Guo S., DiPietro L.A. (2010). Factors affecting wound healing. J. Dent. Res..

[52] White L.A., Mitchell T.I., Brinckerhoff C.E. (2000). Transforming growth factor β inhibitory element in the rabbit matrix metalloproteinase-1 (collagenase-1) gene functions as a repressor of constitutive transcription. Biochim. Biophys. Acta (BBA)-Gene Struct. Expr..

[53] Mohammed H.A., Khan R.A., Abdel-Hafez A.A., Abdel-Aziz M., Ahmed E., Enany S., Mahgoub S., Al-Rugaie O., Alsharidah M., Aly M.S.A. (2021). Phytochemical Profiling, In Vitro and In Silico Anti-Microbial and Anti-Cancer Activity Evaluations and Staph GyraseB and h-TOP-IIβ Receptor-Docking Studies of Major Constituents of Zygophyllum coccineum L. Aqueous-Ethanolic Extract and Its Subsequent Fractions: An Approach to Validate Traditional Phytomedicinal Knowledge. Molecules.

[54] Attallah N.G.M., Negm W.A., Elekhnawy E., Elmongy E.I., Altwaijry N., El-Haroun H., El-Masry T.A., El-Sherbeni S.A. (2021). Elucidation of Phytochemical Content of Cupressus macrocarpa Leaves: In Vitro and In Vivo Antibacterial Effect against Methicillin-Resistant Staphylococcus aureus Clinical Isolates. Antibiotics.

[55] Ibrahim R.S., El-Banna A.A. (2021). Royal jelly fatty acids bioprofiling using TLC-MS and digital image analysis coupled with chemometrics and non-parametric regression for discovering efficient biomarkers against melanoma. RSC Adv..

[56] Owusu E., Ahorlu M.M., Afutu E., Akumwena A., Asare G.A. (2021). Antimicrobial Activity of Selected Medicinal Plants from a Sub-Saharan African Country against Bacterial Pathogens from Post-Operative Wound Infections. Med. Sci..

[57] Wayne A., Clinical and Laboratory Standards Institute (CLSI) (2017). Performance Standards for Antimicrobial Susceptibility Testing.

[58] El-Banna T., Abd El-Aziz A., Sonbol F., El-Ekhnawy E. (2019). Adaptation of Pseudomonas aeruginosa clinical isolates to benzalkonium chloride retards its growth and enhances biofilm production. Mol. Biol. Rep..

[59] Hacioglu M., Oyardi O., Bozkurt-Guzel C., Savage P.B. (2020). Antibiofilm activities of ceragenins and antimicrobial peptides against fungal-bacterial mono and multispecies biofilms. J. Antibiot..

[60] Alotaibi B., Negm W.A., Elekhnawy E., El-Masry T.A., Elseady W.S., Saleh A., Alotaibi K.N., El-Sherbeni S.A. (2021). Antibacterial, Immunomodulatory, and Lung Protective Effects of Boswelliadalzielii Oleoresin Ethanol Extract in Pulmonary Diseases: In Vitro and In Vivo Studies. Antibiotics.

[61] Chan-Zapata I., Canul-Canche J., Fernández-Martín K., Martín-Quintal Z., Torres-Romero J.C., Lara-Riegos J.C., Ramírez-Camacho M.A., Arana-Argáez V.E. (2018). Immunomodulatory effects of the methanolic extract from Pouteria campechiana leaves in macrophage functions. Food Agric. Immunol..

[62] Ezzat M.I., Hassan M., Abdelhalim M.A., El-Desoky A.M., Mohamed S.O., Ezzat S.M. (2021). Immunomodulatory effect of Noni fruit and its isolates: Insights into cell-mediated immune response and inhibition of LPS-induced THP-1 macrophage inflammation. Food Funct..

[63] Elekhnawy E.A., Sonbol F.I., Elbanna T.E., Abdelaziz A.A. (2021). Evaluation of the impact of adaptation of Klebsiella pneumoniae clinical isolates to benzalkonium chloride on biofilm formation. Egypt. J. Med. Hum. Genet..

[64] Tramontina V.A., Machado M.A.N., Filho G.d.R.N., Kim S.H., Vizzioli M.R., Toledo S. (2002). Effect of bismuth subgallate (local hemostatic agent) on wound healing in rats. Histological and histometric findings. Braz. Dent. J..

[65] Chang A.C., Dearman B., Greenwood J.E. (2011). A comparison of wound area measurement techniques: Visitrak versus photography. Eplasty.

[66] Miranda K., Espey M., Wink D. (2001). Unique oxidative mechanisms for the reactive nitrogen oxide species. Nitric Oxide Biol. Chem..

[67] Livak K.J., Schmittgen T.D. (2001). Analysis of relative gene expression data using real-time quantitative PCR and the 2− ΔΔCT method. Methods.

[68] Cakin M.C., Ozdemir B., Kaya-Dagistanli F., Arkan H., Bahtiyar N., Anapali M., Akbas F., Onaran I. (2020). Evaluation of the in vivo wound healing potential of the lipid fraction from activated platelet-rich plasma. Platelets.

