# Supplementary material for: Wound-Healing Potential of Rhoifolin-Rich Fraction Isolated from Sanguisorba officinalis Roots Supported by Enhancing Re-Epithelization, Angiogenesis, Anti-Inflammatory, and Antimicrobial Effects
Source: Pharmaceuticals (Basel). 2022 Jan 31;15(2):178. doi: 10.3390/ph15020178 (PMC8874642; doi:10.3390/ph15020178)
Supplement: Supplementary file 1 [file pharmaceuticals-15-00178-s001.zip › pharmaceuticals-1567946-supplementary.pdf]

Supporting information

Wound Healing Potential of Rhoifolin Rich Fraction Isolated from *Sanguisorba officinalis* Roots Supported by Enhancing Re-Epithelization, Angiogenesis, Anti-Inflammatory, and Antimicrobial Effects

Walaa A. Negm [1,†], Aya H. El-Kadem [2,†], Engy Elekhnawy [3], Nashwah G. M. Attallah [4,*], Gadah Abdulaziz Al-Hamoud [5], Thanaa A. El-Masry [2] and Ahmed Zayed [1,6,*]

[1] Department of Pharmacognosy, Faculty of Pharmacy, Tanta University, Tanta 31527, Egypt; walaa.negm@pharm.tanta.edu.eg (W.A.N.), ahmed.zayed1@pharm.tanta.edu.eg (A.Z)

[2] Department of Pharmacology and Toxicology, Faculty of Pharmacy, Tanta University, Tanta 31527, Egypt; aya.elkadeem@pharm.tanta.edu.eg (A.H.E.-K.); thanaa.elmasri@pharm.tanta.edu.eg (T.A.E.)

[3] Pharmaceutical Microbiology Department, Faculty of Pharmacy, Tanta University, Tanta 31527, Egypt; engy.ali@pharm.tanta.edu.eg (E.E.)

[4] Department of Pharmaceutical Sciences, College of Pharmacy, Princess Nourah bint Abdulrahman University, Riyadh 84428, Saudi Arabia; ngmohamed@pnu.edu.sa (N.G.M.A.)

[5] Department of Pharmacognosy, College of Pharmacy, King Saud University, Riyadh 11495, Saudi Arabia; galhamoud@ksu.edu.sa (G.A.A.H.)

[6] Institute of Bioprocess Engineering, Technical University of Kaiserslautern, Gottlieb-Daimler-Straße 49, 67663 Kaiserslautern, Germany

* Correspondence: ngmohamed@pnu.edu.sa (N.G.M.A.), ahmed.zayed1@pharm.tanta.edu.eg (A.Z)

†These authors contributed equally to this work.

List of Supplemetary Figures

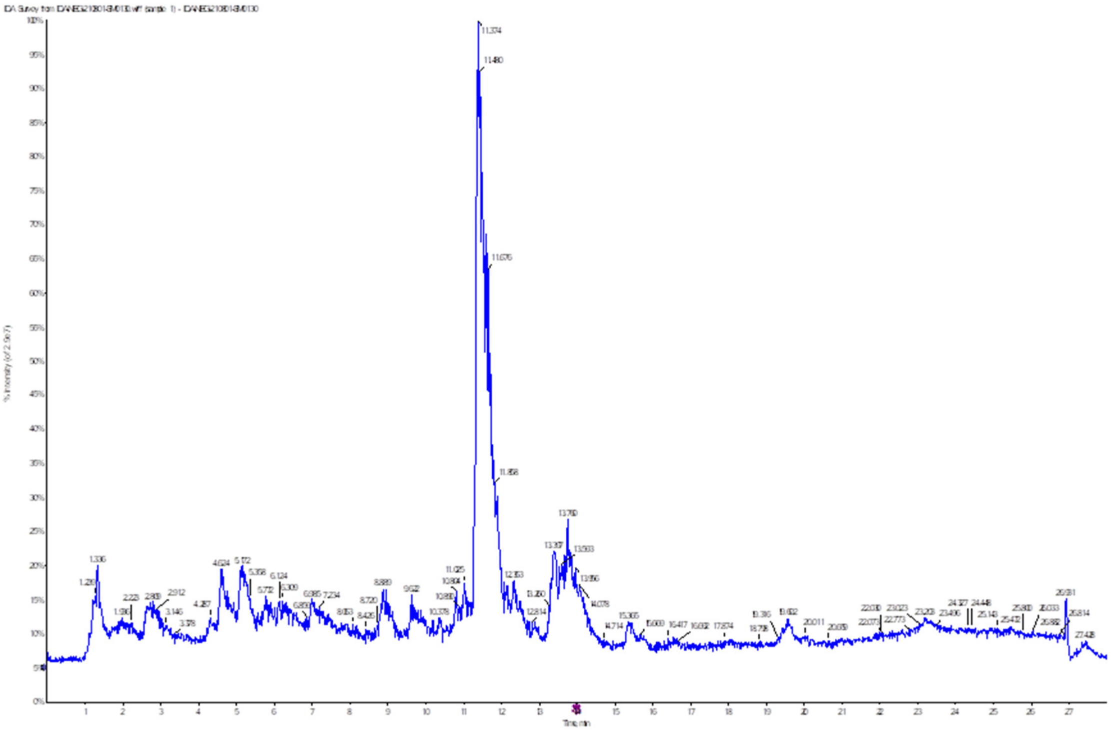

Figure S1: TIC of LC-ESI-MS/MS analysis of *S. officinalis* roots ethanol extract in negative ionization mode

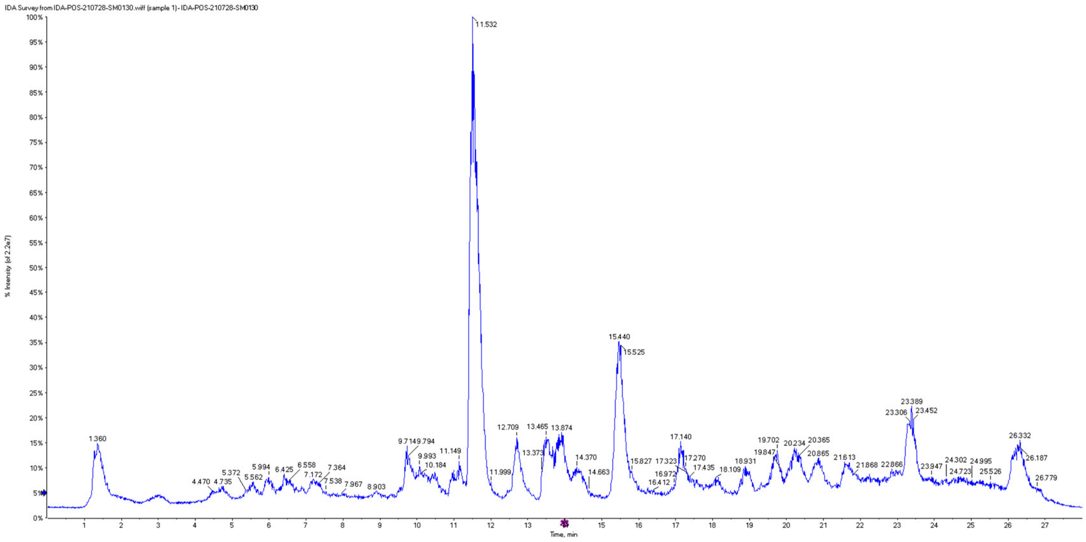

Figure S2: TIC of LC-ESI-MS/MS analysis of *S. officinalis* roots ethanol extract in positive ionization mode

XIC of 343.046 mass

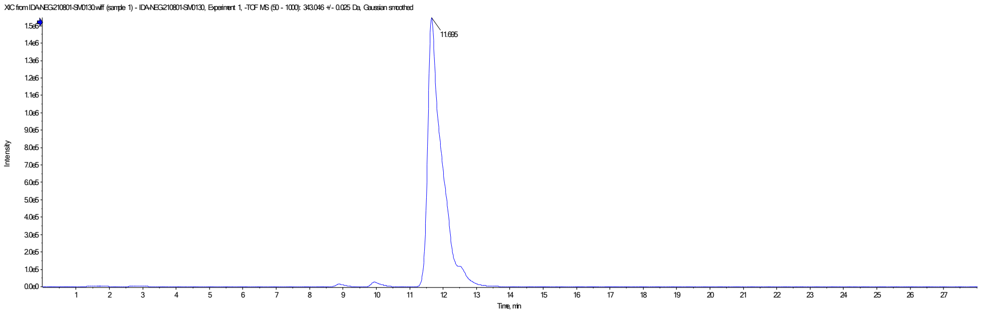

XIC of 423.003 mass

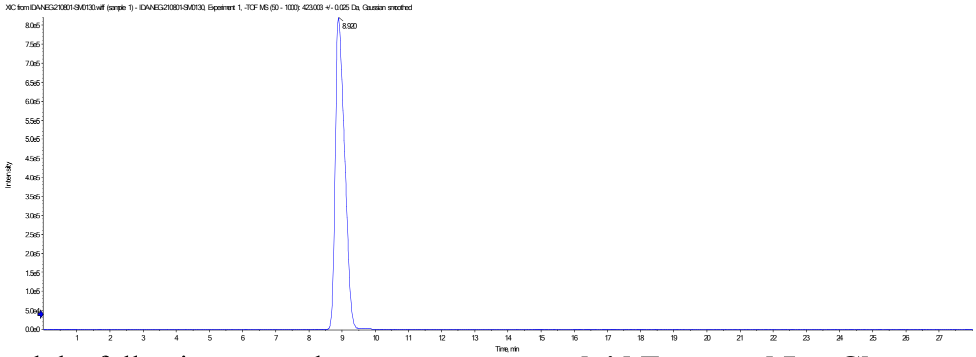

And the following example represents an **overlaid Extracted Ion Chromatograms** for five different masses at different retention times from which we can have an overview of chromatographic separation

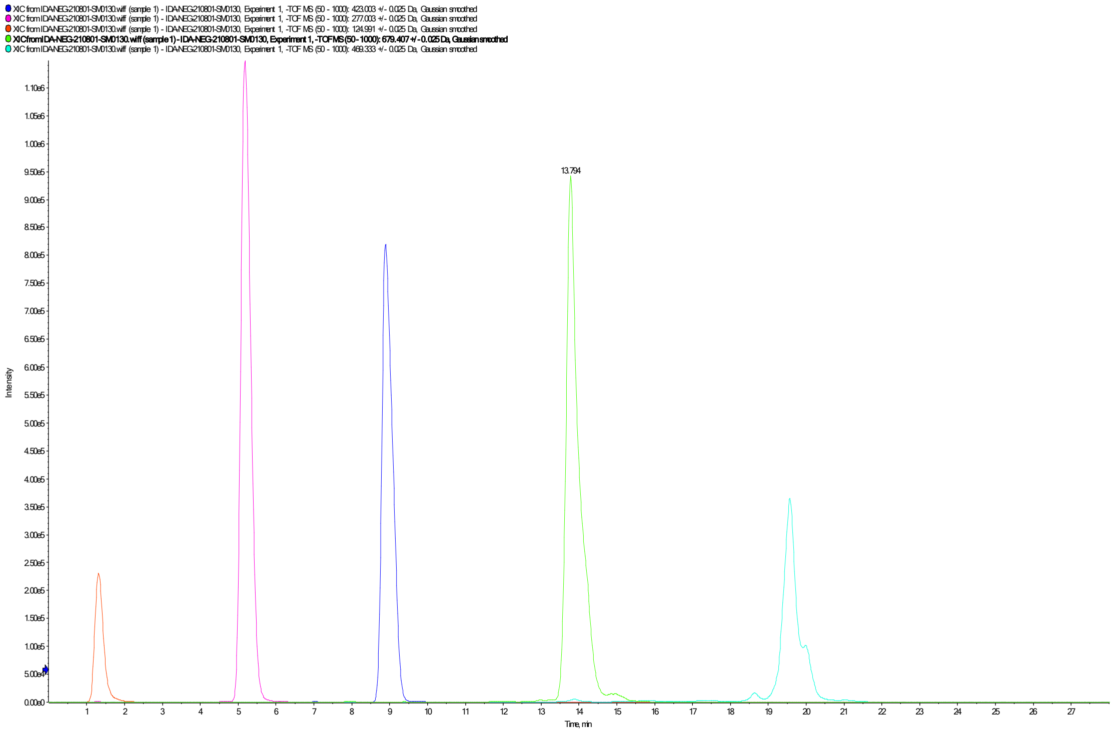

The above conditions are also valid for the other TIC found in **Figure S2:** TIC of LC-ESI-MS/MS analysis of S. *officinalis* roots ethanol extract in positive ionization mode, upon single mass extraction

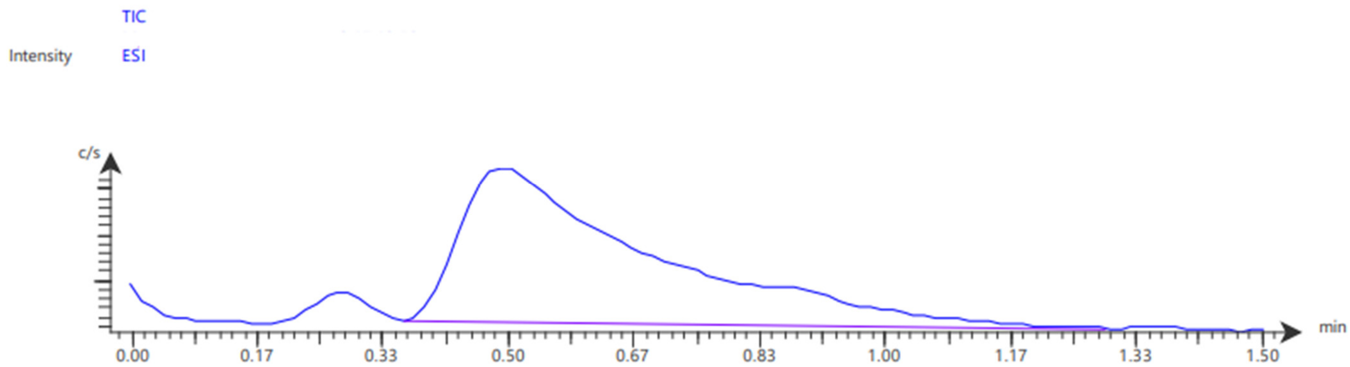

Time (Peak Maximum M:S/Minutes)	Maximum Intensity (c/s)	Time (Peak Centroid M:S/Minutes)	Peak Area	% Peak Area	Peak Resolution	Base Peak Mass (m/z)	Label
0.28	3.263E9	0.28	1.631E10	5.2	4.9	216.1	
0.49	1.7E10	0.52	2.976E11	94.5	13.6	270.1	
1.37	2.041E8	1.37	8.832E8	0.3	7.8	270.2	

Figure S3: Total ion chromatogram (TIC) of rhoifolin rich fraction (RRF) isolated from *S. officinalis* roots ethanol extract

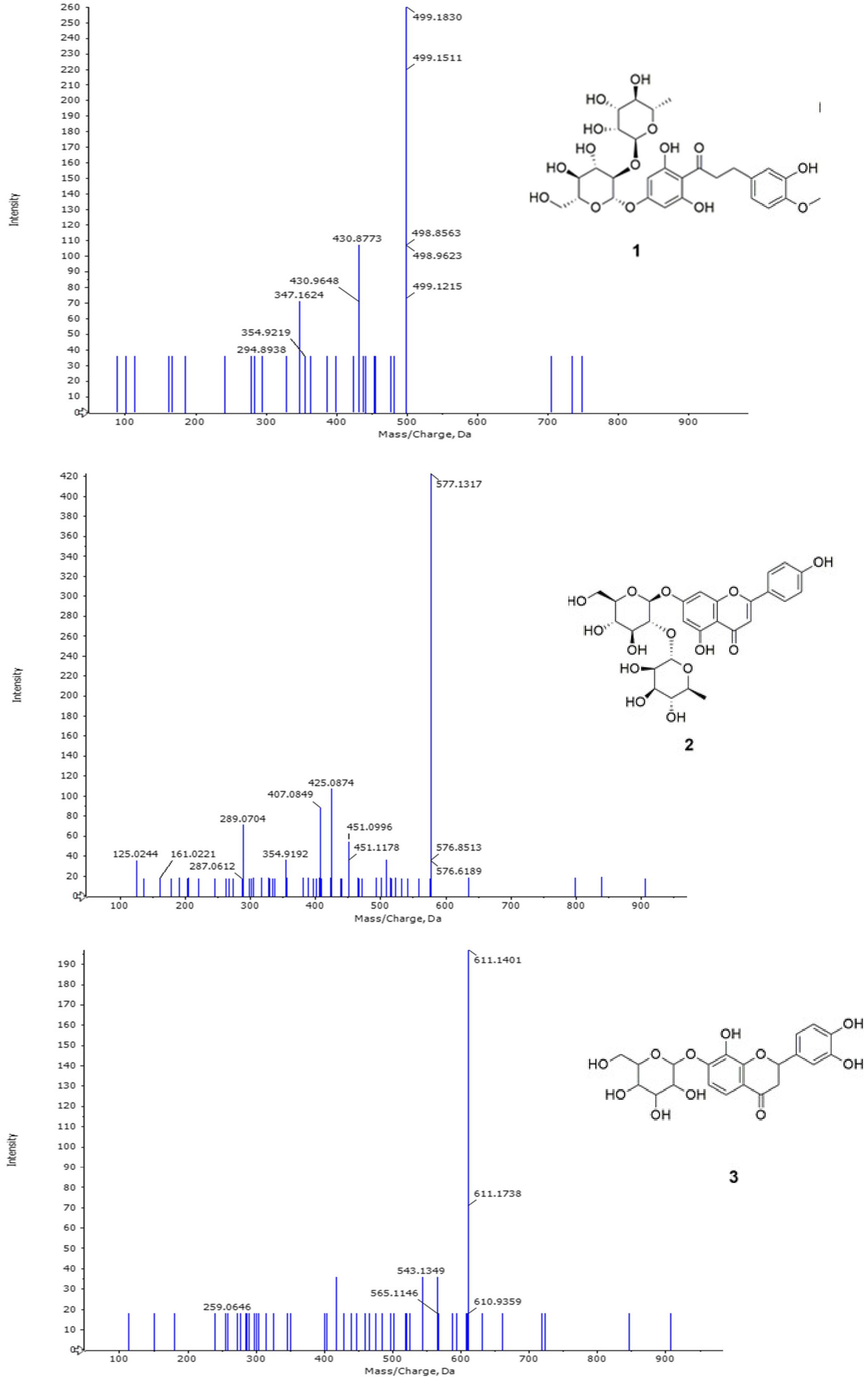

Figure S4: ESI/MS of identified compounds in rhiofolin rich fraction (RRF). The compounds were tentatively identified as neohesperidin dihydrochalcone **(1)**, apigenin 7-*O*-neohesperidoside (Rhoifolin) **(2)**, and isookanin-7-glucoside (flavanomarein) **(3)**.

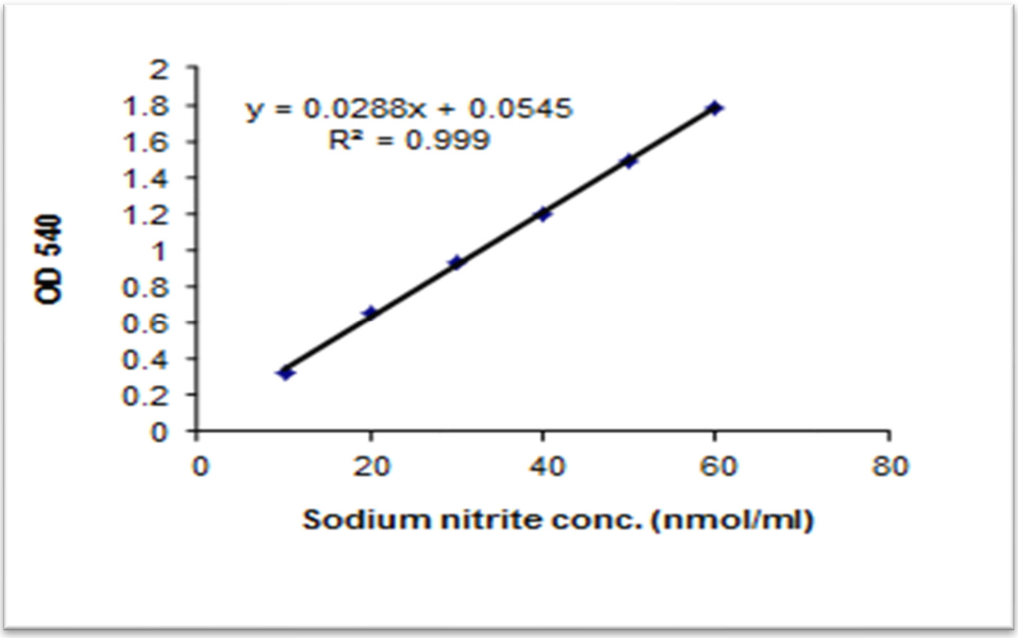

Figure S5: NO standard curve

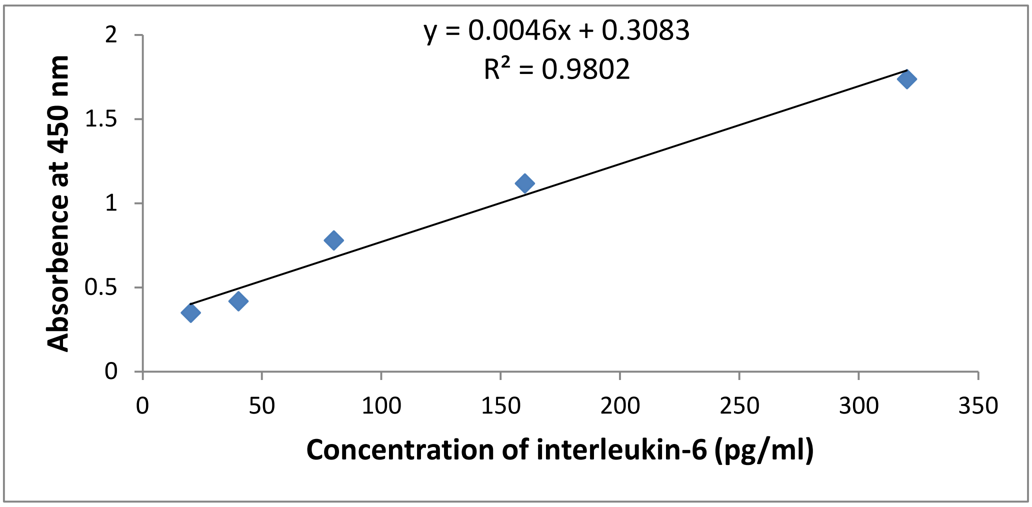

Figure S6: IL-6 standard curve

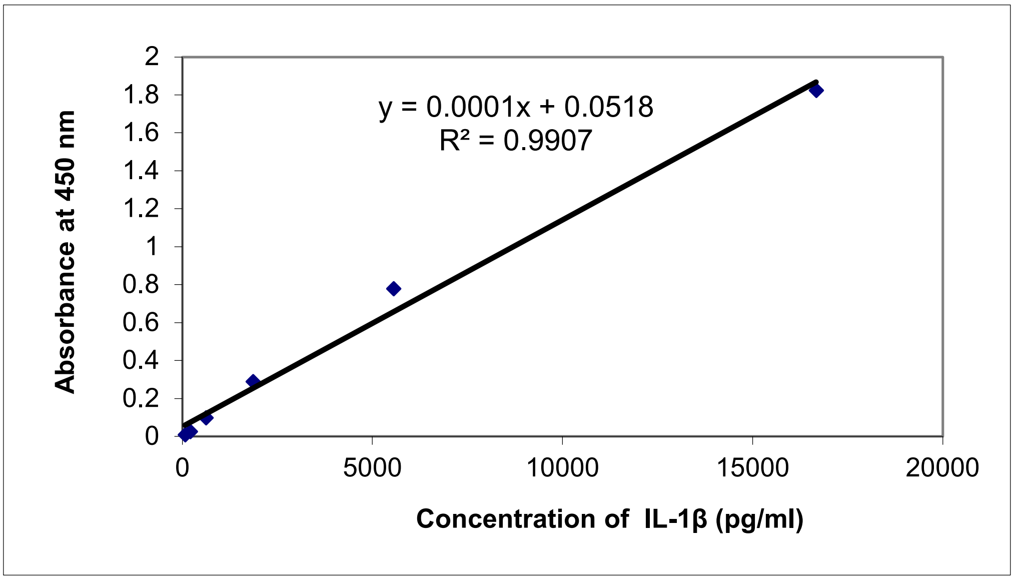

Figure S7: IL-1β standard curve

List of Supplementary Tables

Table S1. MIC values of RRF against the tested *P. aeruginosa* isolates and the level of their biofilm forming ability.

Isolate code	RRF MIC value (µg/mL)	Level of biofilm forming ability*	Isolate code	RRF MIC value (µg/mL)	Level of biofilm forming ability*
P1	128	Strong	P9	128	Weak
P2	64	Strong	P10	256	Weak
P3	256	Strong	P11	128	Moderate
P4	256	Strong	P12	64	Not forming
P5	512	Not forming	P13	128	Weak
P6	64	Weak	P14	256	Weak
P7	64	Moderate	P15	64	Not forming
P8	512	Moderate			

*Based on the results of crystal violet assay

Table S2. MIC values of RRF against the tested *S. aureus* isolates and the level of their biofilm forming ability.

Isolate code	RRF MIC value (µg/mL)	Level of biofilm forming ability*	Isolate code	RRF MIC value (µg/mL)	Level of biofilm forming ability*
S1	128	Strong	S14	128	Not forming
S2	64	Strong	S15	256	Not forming
S3	256	Strong	S16	512	Moderate
S4	256	Strong	S17	64	Moderate
S5	512	Weak	S18	64	Weak

S6	512	Not forming	S19	64	Not forming
S7	64	Moderate	S20	256	Weak
S8	128	Weak	S21	512	Not forming
S9	128	Moderate	S22	256	Moderate
S10	64	Weak	S23	64	Moderate
S11	512	Weak	S24	128	Weak
S12	256	Moderate	S25	256	Moderate
S13	128	Not forming	S26	128	Moderate

*Based on the results of crystal violet assay

Table S3. Sequences of the utilized primers (*in vitro* study)

Gene	Primer sequence*
COX-2	F 5′-TTCAAATGAGATTGTGGGAAAATTGCT-3′<br>R 5′-AGATCATCTCTGCCTGAGTATCTT-3′
iNOS	F 5′-TCTTGGTCAAAGCTGTGCTC-3′<br>R 5′-CATTGCCAAACGTACTGGTC-3′
IL-6	F 5′-AAAGAGGCACTGGCAGAAAA-3′<br>R 5′-AGCTCTGGCTTGTTCCTCAC-3′
TNF-α	F 5'-ATGAGCACTGAAAGCATGATC-3'<br>R 5'-TCACAGGGCAATGATCCCAAAGTAGACCTGCCC-3'
NF-κB	F 5′-GCGGGAGAGGGGATTCCCTGCGGCCCCG-3′<br>R 5′-CGGGGCCGCAGGGAATCCCCTCTCCCGC-3′
GAPDH	F 5'-ACCACAGTCCATGCCATCAC-3'<br>R 5'-TCCACCACCCTGTTGCTGTA-3'

* F stands for forward and R stands for reverse.

Table S4. Forward and reverse primer sequences used in quantitative RT-PCR (*in vivo* study)

Target gene	Probe
VEGF	F 5'-AGGCTGCACCCACGACAGAA-3'<br>R 5'-CTTTGGTCTGCATTCACATC-3'
PDGF-B	F 5′-TGCCAGAGCCTGCTCTTAAC-3′<br>R 5′-GATGCCACGGAGATAAGCGA-3′
Keratinocyte growth factor	F 5′-TCTGTCGAACACAGTGGTACCT-3′<br>R 5′-GTGTGTCCATTTAGCTGATGCAT
Fibronectin	F 5′-GAGCTATCCATTTCACCTTCAGA<br>R 5′-TTGTTCGTAGACACTGGAGAC
MMP-1	F 5′-TGGGATTTCCAAAAGAGGTG<br>R 5′-ACGTGGTTCCCTGAGAAGA
GAPDH	F 5'-CAGCAATGCATCCTGCAC-3'<br>R 5'-GAGTTGCTGTTGAAGTCACAGG-3'
